# Identification and transcriptomic assessment of latent profile pediatric septic shock phenotypes

**DOI:** 10.1186/s13054-024-05020-z

**Published:** 2024-07-17

**Authors:** Mihir R. Atreya, Min Huang, Andrew R. Moore, Hong Zheng, Yehudit Hasin-Brumshtein, Julie C. Fitzgerald, Scott L. Weiss, Natalie Z. Cvijanovich, Michael T. Bigham, Parag N. Jain, Adam J. Schwarz, Riad Lutfi, Jeffrey Nowak, Neal J. Thomas, Michael Quasney, Mary K. Dahmer, Torrey Baines, Bereketeab Haileselassie, Andrew J. Lautz, Natalja L. Stanski, Stephen W. Standage, Jennifer M. Kaplan, Basilia Zingarelli, Rashmi Sahay, Bin Zhang, Timothy E. Sweeney, Purvesh Khatri, L. Nelson Sanchez-Pinto, Rishikesan Kamaleswaran

**Affiliations:** 1 Division of Critical Care Medicine, MLC2005, Cincinnati Children’s Hospital Medical Center, 3333 Burnet Avenue, Cincinnati, OH 45229 USA; 2 Department of Pediatrics, University of Cincinnati College of Medicine, Cincinnati, OH 45627 USA; 3 Department of Biomedical Informatics, Emory University School of Medicine, Atlanta, GA USA; 4 Stanford Institute for Immunity, Transplantation and Infection, Stanford University School of Medicine, Stanford, CA USA; 5 Center for Biomedical Informatics Research, Department of Medicine, Stanford University School of Medicine, Stanford, CA 94305 USA; 6 Inflammatix, Sunnyvale, CA 94085 USA; 7 Children’s Hospital of Philadelphia, Philadelphia, PA 19104 USA; 8 Nemours Children’s Health, Wilmington, DE 19803 USA; 9 UCSF Benioff Children’s Hospital Oakland, Oakland, CA 94609 USA; 10 Akron Children’s Hospital, Akron, OH 44308 USA; 11 Texas Children’s Hospital, Baylor College of Medicine, Houston, TX 77030 USA; 12 Children’s Hospital of Orange County, Orange, CA 92868 USA; 13 Riley Hospital for Children, Indianapolis, IN 46202 USA; 14 Children’s Hospital and Clinics of Minnesota, Minneapolis, MN 55404 USA; 15 Penn State Hershey Children’s Hospital, Hershey, PA 17033 USA; 16 C.S Mott Children’s Hospital, University of Michigan, Ann Arbor, MI 48109 USA; 17 University of Florida Health Children’s Hospital, Gainesville, FL 32610 USA; 18 Lucile Packard Children’s Hospital Stanford, Palo Alto, CA 94304 USA; 19 Division of Biostatistics and Epidemiology, Cincinnati Children’s Hospital Medical Center, Cincinnati, OH 45229 USA; 20 Department of Pediatrics, Northwestern University Feinberg School of Medicine, Chicago, IL 60611 USA; 21 Department of Health and Biomedical Informatics, Northwestern University Feinberg School of Medicine, Chicago, IL 60611 USA; 22 Department of Biomedical Informatics, Emory University School of Medicine, Atlanta, GA 30322 USA; 23 Department of Biomedical Engineering, Georgia Institute of Technology, Atlanta, GA 30322 USA

**Keywords:** Sepsis, Precision medicine, Endotype, Phenotype

## Abstract

**Background:**

Sepsis poses a grave threat, especially among children, but treatments are limited owing to heterogeneity among patients. We sought to test the clinical and biological relevance of pediatric septic shock subclasses identified using reproducible approaches.

**Methods:**

We performed latent profile analyses using clinical, laboratory, and biomarker data from a prospective multi-center pediatric septic shock observational cohort to derive phenotypes and trained a support vector machine model to assign phenotypes in an internal validation set. We established the clinical relevance of phenotypes and tested for their interaction with common sepsis treatments on patient outcomes. We conducted transcriptomic analyses to delineate phenotype-specific biology and inferred underlying cell subpopulations. Finally, we compared whether latent profile phenotypes overlapped with established gene-expression endotypes and compared survival among patients based on an integrated subclassification scheme.

**Results:**

Among 1071 pediatric septic shock patients requiring vasoactive support on day 1 included, we identified two phenotypes which we designated as *Phenotype 1* (19.5%) and *Phenotype 2* (80.5%). Membership in *Phenotype 1* was associated *with* ~ fourfold adjusted odds of complicated course relative to *Phenotype 2*. Patients belonging to *Phenotype 1* were characterized by relatively higher Angiopoietin-2/Tie-2 ratio, Angiopoietin-2, soluble thrombomodulin (sTM), interleukin 8 (IL-8), and intercellular adhesion molecule 1 (ICAM-1) and lower Tie-2 and Angiopoietin-1 concentrations compared to *Phenotype 2*. We did not identify significant interactions between phenotypes, common treatments, and clinical outcomes. Transcriptomic analysis revealed overexpression of genes implicated in the innate immune response and driven primarily by developing neutrophils among patients designated as *Phenotype 1*. There was no statistically significant overlap between established gene-expression endotypes, reflective of the host adaptive response, and the newly derived phenotypes, reflective of the host innate response including microvascular endothelial dysfunction. However, an integrated subclassification scheme demonstrated varying survival probabilities when comparing patient endophenotypes.

**Conclusions:**

Our research underscores the reproducibility of latent profile analyses to identify pediatric septic shock phenotypes with high prognostic relevance. Pending validation, an integrated subclassification scheme, reflective of the different facets of the host response, holds promise to inform targeted intervention among those critically ill.

**Graphical abstract:**

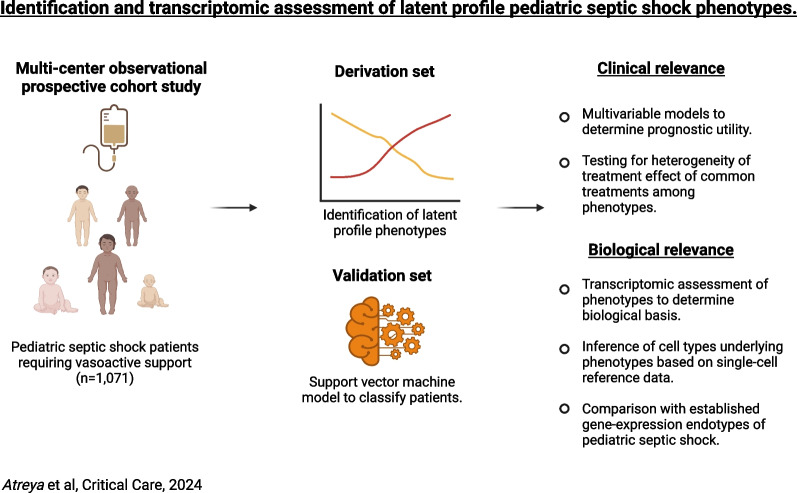

**Supplementary Information:**

The online version contains supplementary material available at 10.1186/s13054-024-05020-z.

## Introduction

Sepsis is defined as life-threatening organ dysfunction caused by a dysregulated host response to an infection. It represents a major public health problem, especially among children, where it affects an estimated 20 million each year worldwide [1] and is the leading cause of under-5 mortality [2]. Yet, despite numerous trials, sepsis care remains limited to early antibiotics and intensive organ support. This lack of therapeutic efficacy has been attributed to the heterogeneity among critically ill patients [3]. Thus, reproducible strategies that identify clinically and biologically relevant subclasses are necessary to facilitate targeted approaches to improve patient outcomes [4].

Gene-expression profiling of whole blood has been used to identify sepsis subclasses [5–9]. Among children, *Wong* and colleagues used a 100 gene-expression panel, to identify pediatric septic shock *Endotypes*—*A* and *B* with prognostic value; assignment to *Endotype A* was associated with a nearly threefold increased risk of mortality, relative to those with *Endotype B* [10]. Subsequently, these endotypes were shown to demonstrate a differential response to corticosteroids in observational studies, with patients classified as *Endotype A* having a fourfold increase in mortality with use of adjunctive corticosteroid use, relative to patients with *Endotype B* [11]. Similar strategies have been deployed among adults yielding analogous results [12].

Concomitantly, a decade ago, *Calfee* et al. leveraged latent class analyses of clinical, laboratory, and biomarker data to identify two phenotypes of acute respiratory distress syndrome (ARDS). The *hyperinflammatory* group was characterized by worse outcomes, relative to those without this phenotype [13]. Of note, these phenotypes have demonstrated heterogeneity in treatment effect (HTE) in response to several interventions in secondary analyses of ARDS trials [13, 14], and corticosteroids among critically ill COVID-19 patients [15]. More recently, *Dahmer* et al. and others have shown reproducibility and prognostic utility of this approach among children with ARDS [16, 17]. Lastly, using similar approaches, S*inha* et al. recently published on molecular phenotypes among adults with sepsis [18]. To the best of our knowledge, no study to date has identified latent profile phenotypes, inclusive of biomarker data, among critically ill children with sepsis.

In the current study, we sought to derive and internally validate pediatric septic shock phenotypes using latent profile analyses in our multi-center prospective observational cohort and to establish their prognostic value. We sought to test interactions between phenotypes and common treatments on patient outcomes. To establish their biological significance, we conducted transcriptomic analyses in a subset of the cohort to identify differentially expressed genes and infer cell subpopulations linked to phenotypes. Lastly, we compared the overlap between previously established gene-expression endotypes of pediatric septic shock and newly identified latent profile phenotypes. We tested the hypothesis that integrating endotype and phenotype assignment could provide a refined framework for the subclassification of critically ill children.

## Methods

### Study design and patient selection

Our ongoing prospective observational cohort study of pediatric septic shock has been extensively detailed previously [11, 19–21]. All study procedures involving human participants were per the ethical standards of the institutional review boards of participating institutions and consistent with the 1964 Helsinki Declaration and its later amendments or comparable ethical standards. Briefly, children ≤ 18 years of age were enrolled after informed consent was obtained from parents or legal guardians. Inclusion criteria for study enrollment were all patients meeting consensus criteria for pediatric septic shock [22] recruited between 2003 and 2023 from 13 pediatric intensive care units (PICUs) in the U.S. Blood was collected from consenting participants within 24 h of meeting enrollment criteria (day 1). Patients who did not require any vasoactive support were excluded from the current analyses. The primary outcome of interest was complicated course—a composite endpoint of death by or presence of ≥ 2 organ dysfunctions on day 7 after study enrollment [20]. Secondary outcomes included 7- and 28-day mortality.

### Data imputation

We excluded variables with ≥ 40% missingness of data. Among those with < 40% missingness, we used python package “Datawig” which uses deep learning feature extraction with automatic hyperparameter tuning to impute missing value [23]. Additional methodological details are presented in the Online Supplement.

### Derivation set

We randomly split patients in the cohort into derivation (60%) and hold-out internal validation (40%) sets. We used R package “mclust” (v.6.0.0) to perform latent profile analyses (LPA)—a Gaussian Finite Mixture Modeling approach– using clinical, laboratory, and biomarker variables collected on day 1 of septic shock. Briefly, we included deviation of vital signs from the median values for age and sex during health. Laboratory data were obtained at the discretion of treating physicians. The most extreme value for the day were included for these variables. Biomarker data were previously measured using multiplex Luminex assays in serum collected on day 1 [20, 24]. Additional methodological details are presented in the Online Supplement.

### Validation set

The phenotype assignments in the derivation set were used to train a support vector machine (SVM) classifier, which was used to assign phenotypes in the validation set using the same set of variables used in the LPA model. We compared patient demographics, characteristics, outcomes in the derivation and validation sets to determine clinical relevance of assigned phenotypes. In sensitivity analyses, we compared biomarkers among identified phenotypes in the validation dataset after exclusion of imputed data to ensure validity and biological relevance of phenotypes.

### Transcriptomic analyses

Bulk messenger RNA sequencing data was available from a subset of the cohort recruited between 2019 and 2023 from day 1 biospecimens. We used DESeq2 (v.1.38.3) to identify differentially expressed genes (DEGs) between the latent profile phenotypes. DEGs were selected based on ≥ log2 fold change value cutoff of ± 0.25, and adjusted *p* value of 0.05. We conducted Reactome pathway analyses [25] using “ReactomePA” package with a Benjamin Hochberg false discovery rate (FDR) < 0.05 to identify enriched biological pathways.

### Inference of cell types underlying phenotypes

We sought to gain granular insight at a single-cell level into immune cell subpopulations associated with latent profile phenotypes. To achieve this, we used a publicly available single-cell RNA sequencing dataset comprised of critically ill adults with sepsis published by *Kwok* et al. [26] We calculated a composite gene score as the geometric mean of overexpressed genes minus the geometric mean of under-expressed genes using published methods [27], identified through DEG analyses comparing latent profile phenotypes and available in the single-cell dataset. We mapped the scaled composite score against the Uniform Manifold Approximation and Projection (UMAP) of the single-cell dataset to infer cell types driving biological differences between phenotypes.

### Comparison with established gene-expression pediatric septic shock endotypes

A subset of patients in the cohort had existing assignments as *Endotypes A* or *B* based on historical data using a 100-gene panel on the Nanostring nCounter platform. Briefly, image analysis of gene-expression mosaics were previously used to assign pediatric septic shock endotypes, with *Endotype A* being characterized by a repressed adaptive immune response and glucocorticoid signaling, relative to *Endotype B* [11].

### Statistical analyses

Minitab (PA, USA) and R were used for statistical analyses. GraphPad (CA, USA) and R were used to generate figures. We assessed differences in demographic and clinical characteristics between groups by non-parametric Kruskal–Wallis tests for continuous variables and χ^2^ tests for categorical variables. Multivariable logistic regression models were used to assess the association between phenotype and outcomes of interest and adjusted for era of enrollment (2013–2023 vs. 2003–2012), patient age, pediatric risk of mortality score (PRISM III) [28], presence of comorbidity, and immunocompromised status. We used inverse probability treatment weighting (IPTW) to test the effect of common sepsis treatments on the odds of complicated course among latent profile phenotypes accounting for the effect of multiple confounding variables [29]. Treatments tested included use of > 100 ml/kg versus < 100 ml/kg fluid resuscitation, ≥ 2 versus < 2 antimicrobials, ≥ 2 versus < 2 vasoactive medications on day 1, and corticosteroid use. For IPTW models, we adjusted for age, PRISM-III score, day 1 vasoactive inotropic score (VIS), presence of comorbidity and immunocompromised status. Interaction *p* values for overall effect were used to test for heterogeneity of treatment effect (HTE) across latent profile phenotypes on complicated course. The Pearson χ^2^ test was used to test the overlap between established gene-expression endotypes and latent profile phenotypes. Kaplan Meier curves were used to estimate differences in survival comparing endotypes, phenotypes, and an integrated subclass assignment scheme where we considered outputs of both these approaches. Cox proportional hazard ratio of 28-day mortality among subclasses was compared in reference to the endophenotype with the lowest 28-day mortality. A two-tailed *p* value < 0.05 was used to test statistical significance, unless otherwise specified.

## Results

**Fig. 1:**
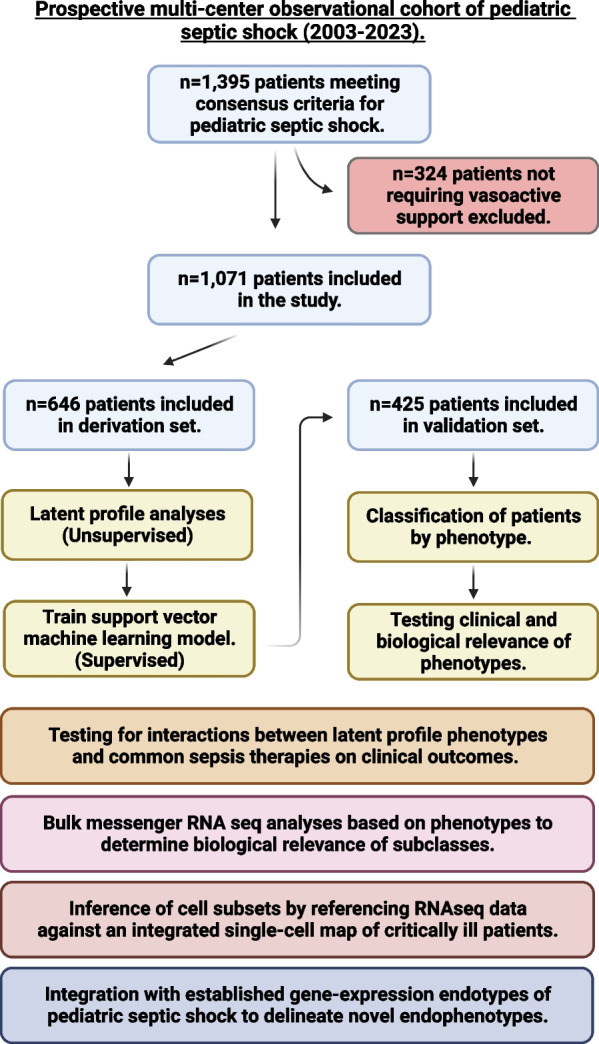
Overview of study including inclusion and exclusion criteria, number of patients across the derivation and validation set, and various analytic approaches used to characterize latent profile phenotypes of pediatric septic shock

**Fig. 2:**
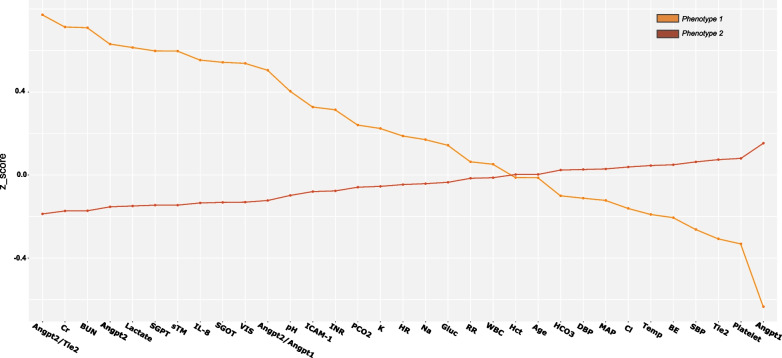
Standardized mean (z-scores) for continuous class predicting variables in the derivation set by latent profile is shown on the y-axis. The predictor variables are sorted on the x-axis from left to right in descending order of difference between the *Phenotype 1* (shown in orange) and *Phenotype 2* (shown in brown) phenotypes. Angpt2/Tie-2: Angiopoietin-2/Tie-2 ratio; Cr: Creatinine; BUN: blood urea nitrogen; Angpt-2: Angiopoietin-2; Lactate: Serum lactate; SGPT: serum glutamic pyruvic transaminase; sTM: soluble Thrombomodulin; IL-8: Interleukin-8; SGOT: serum glutamic-oxaloacetic transaminase; VIS: Max vasoactive inotropic score on day 1; Angpt-2/Angpt-1: Angiopoietin-2/Angiopoietin-1 ratio; pH; ICAM-1: Intercellular adhesion molecule 1; INR: international normalized ratio; PCO2: partial pressure of carbon dioxide; K: potassium; HR: deviation from age and sex normalized heart rate; Na: Sodium; Gluc: Glucose; RR: respiratory rate; WBC: white blood cell count; HCt: hematocrit; Age: age in years; HCO3: serum bicarbonate; DBP: diastolic blood pressure; MAP: mean arterial pressure; Cl: serum chloride; Temp: Temperature; BE: base excess; SBP: systolic blood pressure; Tie-2: tyrosine kinase with immunoglobulin-like loops and epidermal growth factor homology domains-2; Platelet: platelet count; Angpt-1: Angiopoietin-1

The overview of the study and analyses is detailed in Fig. 1. A total of 1,395 patients met the inclusion criteria for the study of whom we excluded 324 patients who did not receive any vasoactive support. The median age of the patients included in the study (n = 1071) was 5.3 years (quartile 1: 1.7; quartile 3: 11.0 years). The derivation set was comprised of 646 patients and the hold-out validation set included 425 patients. Latent profile analyses in the derivation set revealed two phenotypes. Differences in standardized variables between the two phenotypes are shown in Fig. 2. One of the phenotypes (n = 126, 19.5%) was characterized by a relatively higher lactate, serum creatinine, blood urea nitrogen (BUN), and international normalized ratio (INR), and lower platelet counts, which we designated as *Phenotype 1.* Patients in this group had relatively higher Angiopoietin-2/Tie-2 ratio, Angiopoietin-2, soluble thrombomodulin (sTM), interleukin 8 (IL-8), and intercellular adhesion molecule 1 (ICAM-1) and lower Tie-2 and Angiopoietin-1 concentrations. We labeled the remaining patients (n = 520, 80.5%), characterized by the absence of such features, as *Phenotype 2*.

Table 1 shows the comparisons between phenotypes in the derivation and validation sets—the latter based on the assignments of our SVM classifier. There were no differences in age and sex comparing phenotypes. Although patients who were *Phenotype 1* were more likely to have had a history of oncologic disease or bone marrow transplantation than *Phenotype 2* in the derivation set, there were no statistically significant differences in the validation set. Patients with *Phenotype 1* had a trend toward higher rates of positive blood cultures compared to patients with *Phenotype 2* in the derivation set (26.2% vs. 19.2%, *p* = 0.08); this reached statistical significance in the validation set (33.8% vs. 20.6%, *p* = 0.016). However, there were no significant differences in the type of pathogen based on culture. Patients with *Phenotype 1* had higher baseline illness severity and significantly worse clinical outcomes in the derivation and validation sets. Finally, patients with *Phenotype 1* were more likely to have been prescribed corticosteroids by treating physicians, relative to those in *Phenotype 2*.

**Table 1:** Demographics, patient characteristics, and clinical outcomes among pediatric septic shock latent profile phenotypes in the derivation and validation sets

	Derivation set (n = 646)		*p* value	Validation set (n = 425)		*p* value
	*Phenotype 1* (n = 126)	*Phenotype 2* (n = 520)		*Phenotype 1* (n = 71)	*Phenotype 2* (n = 354)	
Age (years)	4.7 (1.3, 13.7)	5.4 (1.8, 10.8)	0.698	6.2 (1.8, 14.0)	5.5 (1.8, 10.4)	0.480
Sex (female)	57 (45.2%)	246 (47.3%)	0.676	39 (54.9%)	174 (49.2%)	0.374
Race			0.924			0.439
White or Caucasian	89 (70.7%)	376 (72.3%)		55 (77.4%)	263 (74.3%)	
Black or African American	16 (12.7%)	64 (12.3%)		6 (8.4%)	49 (13.8%)	
Other	21 (16.7%)	80 (15.4%)		10 (14.1%)	42 (11.9%)	
Ethnicity			0.214			0.063
Hispanic or Latino	12 (9.5%)	71 (13.6%)		3 (4.2%)	41 (11.6%)	
Non-Hispanic	114 (90.5%)	449 (86.4%)		68 (95.7%)	313 (88.4%)	
Culture						
Any positive culture	71 (56.4%)	309 (59.4%)	0.529	44 (61.9%)	198 (55.9%)	0.348
Pulmonary	23 (18.2%)	133 (25.6%)		13 (18.3%)	68 (19.2%)	
Extra-pulmonary	48 (38.1%)	175 (33.6%)		31 (43.7%)	130 36.7%)	
Positive blood culture	33 (26.2%)	100 (19.2%)	0.083	24 (33.8%)	73 (20.6%)	0.016
Pathogen type			0.577			0.467
Gram positive	26 (36.6%)	121 (39.2%)		18 (40.9%)	78 (39.4%)	
Gram negative	28 (39.4%)	122 (39.4%)		17 (38.6%)	88 (44.4%)	
Viral	7 (9.8%)	38 (12.3%)		3 (6.8%)	16 (8.1%)	
Fungal	7 (9.8%)	15 (4.8%)		4 (9.0%)	6 (13.6%)	
Mixed	3 (4.2%)	13 (4.2%)		2 (4.5%)	8 (4.1%)	
Comorbidity						
Heart disease	9 (7.1%)	35 (6.7%)	0.869	4 (5.6%)	24 (6.8%)	0.722
Lung disease	12 (9.5%)	50 (9.6%)	0.975	7 (9.8%)	22 (6.2%)	0.281
Neurologic disease	10 (7.9%)	107 (20.6%)	0.001	9 (12.7%)	67 (18.9%)	0.194
Kidney disease	19 (15.1%)	13 (2.5%)	0.001	5 (7.0%)	10 (2.8%)	0.079
Liver disease	10 (7.9%)	25 (4.8%)	0.164	12 (16.9%)	28 (7.9%)	0.018
Solid organ transplant	5 (4.0%)	13 (2.5%)	0.369	4 (5.6%)	16 (4.5%)	0.686
Oncologic disease	26 (20.6%)	56 (10.8%)	0.003	11 (15.5%)	42 (11.9%)	0.398
Bone marrow transplant	17 (13.5%)	22 (4.3%)	< 0.001	9 (12.8%)	29 (8.2%)	0.227
PRISM III	16 (9, 24)	11 (6, 16)	< 0.001	16 (11, 23)	10 (6, 15)	< 0.001
Day 1 VIS	30 (10, 100)	15 (7, 40)	< 0.001	40 (13, 150)	16 (8, 31)	< 0.001
Day 1 P/F < 250	31 (24.6%)	118 (22.7%)	0.648	23 (32.4%)	69 (19.5%)	< 0.016
PICU LOS	7 (2, 15)	6 (2, 12)	0.673	7 (2, 14)	5 (2, 11)	0.815
PICU Free days	22 (12, 26)	22 (16, 26)	0.668	21 (14, 26)	23 (17, 26)	0.804
Hospital LOS	14 (5, 28)	13 (7, 27)	0.955	15 (3, 28)	14 (7, 26)	0.441
7-day mortality	31 (24.6%)	27 (5.2%)	< 0.001	20 (28.2%)	19 (5.4%)	< 0.001
28-day mortality	41 (32.5%)	46 (8.9%)	< 0.001	25 (35.2%)	30 (8.5%)	< 0.001
Complicated course	75 (59.5%)	138 (26.5%)	< 0.001	48 (67.6%)	96 (27.1%)	< 0.001
Cardiac arrest	67 (53.2%)	76 (14.6%)	< 0.001	38 (53.5%)	55 (15.5%)	< 0.001
Day 7 Cardiovascular dysfunction	54 (42.8%)	85 (16.4%)	< 0.001	36 (50.7%)	71 (20.1%)	< 0.001
Day 7 Respiratory Dysfunction	72 (57.2%)	170 (32.7%)	< 0.001	46 (64.8%)	120 (33.9%)	< 0.001
Day 7 Kidney Dysfunction	64 (50.8%)	104 (20.0%)	< 0.001	42 (59.2%)	68 (19.2%)	< 0.001
Day 7 Neuro Dysfunction	27 (21.4%)	24 (4.6%)	< 0.001	19 (26.8%)	19 (5.4%)	< 0.001
Day 7 Hematologic Dysfunction	59 (46.8%)	79 (15.2%)	< 0.001	36 (50.7%)	48 (13.6% 0	< 0.001
Day 7 Hepatic Dysfunction	50 (39.7%)	57 (11.0%)	< 0.001	34 (47.9%)	31 (8.8%)	< 0.001
Day 7 Vasoactive support^†^	28/70 (40.0%)	55/278 (19.7%)	< 0.001	15/39 (38.4%)	40/173 (23.1%)	< 0.001
Day 7 Mechanical ventilation^†^	51/70 (72.8%)	164/278 (58.9%)	0.033	30/39 (76.9%)	101/173 (58.3%)	0.031
Day 7 CRRT^†^	27/70 (38.6%)	22/278 (7.9%)	< 0.001	10/39 (25.6%)	12/173 (6.9%)	< 0.001
Day 1–7% positive fluid balance	6.6 (1.9, 16.6%)	4.9 (0.0, 11.7)	0.016	8.3 (1.7, 17.8)	4.9 (0.7, 11.6)	0.008
Any ECMO	2 (1.6%)	1 (0.2%)	0.039	1 (1.4%)	1 (0.3%)	0.345
Corticosteroids	82 (65.1%)	279 (53.7%)	0.020	53 (74.7%)	187 (52.8%)	< 0.001

PRISM III, Pediatric risk of mortality score-III; VIS, vasoactive inotropic score; P/F, PaO2/FiO2 ratio; LOS, length of stay; CRRT, Continuous renal replacement therapy; ECMO: Extracorporeal membrane oxygenation. ✝Indicates data only among patients alive and remaining in the PICU on Day 7 after enrollment

Table 2 shows the results of multi-variable logistic regression testing the association between latent profile phenotypes and outcomes. Patients belonging to *Phenotype 1* had a nearly fourfold higher odds of complicated course (adj. OR 3.9, 95% CI 2.8–5.5, *p* < 0.001) relative to *Phenotype 2*. In addition, these patients had an over fivefold higher odds of 7-day mortality (adj. OR 5.6, 95% CI 3.6–8.6, *p* < 0.001) and over fourfold higher odds of 28-day mortality (adj. OR 4.4, 95% CI 3.0–6.4, *p* < 0.001). Table 3 shows the results of unadjusted, IPTW adjusted associations, and overall interaction between latent profile phenotypes and common sepsis therapies on odds of complicated course. Patients with *Phenotype 1* were more likely to have received ≥ 100 ml/kg of fluid on day 1 of PICU admission, ≥ 2 antimicrobials, ≥ 2 vasoactive agents, and corticosteroids, with commensurately worse outcomes, relative to those belonging to *Phenotype 2*. We did not identify any significant heterogeneity of treatment effect on outcomes with one exception. Patients belonging to *Phenotype 1* who received ≥ 2 antimicrobial therapies had a higher odds of complicated course in comparison with *Phenotype 2* who had a lower odds of the outcome (interaction *p* value 0.016).

**Table 2:** Logistic regression analyses to test association between latent profile phenotypes across derivation and validation sets and pediatric septic shock outcomes

Variable	Unadjusted OR	Adjusted OR*	*p* value
Complicated Course			
*Phenotype 1* (relative to *Phenotype 2*)	4.8 (3.5, 6.6)	3.9 (2.8, 5.5)	< 0.001
7-day mortality			
*Phenotype 1* (relative to *Phenotype 2*)	6.7 (4.4, 10.2)	5.6 (3.6, 8.6)	< 0.001
28-day mortality			
*Phenotype 1* (relative to *Phenotype 2*)	5.6 (3.9, 8.1)	4.4 (3.0, 6.5)	< 0.001

*All models adjusted for era of enrollment (2013–2023 vs. 2003–2013), age, PRISM III illness severity score, co-morbidity, and immunocompromised status

**Table 3:** Unadjusted, inverse probability treatment weighting (IPTW) adjusted association, and overall interaction between latent profile phenotypes and common sepsis treatments on odds of complicated course in the cohort

Treatment effect	*Phenotype 1*		*Phenotype 2*		P interaction
	OR (95% CI)	*p* value	OR (95% CI)	*p* value	
> 100 ml/kg fluid					
Unadjusted	2.67 (1.47–4.86)	0.0013	1.91 (1.39–2.63)	< 0.0001	
IPTW Adjusted	2.93 (1.95–4.38)	< 0.0001	1.75 (1.40–2.17)	< 0.0001	0.184
≥ 2 Antimicrobials					
Unadjusted	3.53 (1.34–9.31)	0.0108	0.91 (0.55–1.52)	0.7294	
IPTW Adjusted	3.02 (2.00–4.56)	< 0.0001	0.82 (0.66–1.01)	0.0577	0.016
≥ 2 Vasoactives					
Unadjusted	2.44 (1.35–4.41)	0.0031	1.91 (1.41–2.59)	< 0.0001	
IPTW Adjusted	1.63 (1.07–2.48)	0.0218	1.66 (1.34–2.05)	< 0.0001	0.624
Corticosteroids use					
Unadjusted	2.88 (1.55–5.37)	0.0008	1.7 (1.25–2.31)	0.0007	
IPTW Adjusted	2.55 (1.70–3.85)	< 0.0001	1.49 (1.20–1.85)	0.0003	0.102

*Inverse probability treatment weighting (IPTW) models adjusted for age, PRISM-III score, vasoactive inotropic score (VIS), co-morbidity, and immunocompromised status

**Fig. 3:**
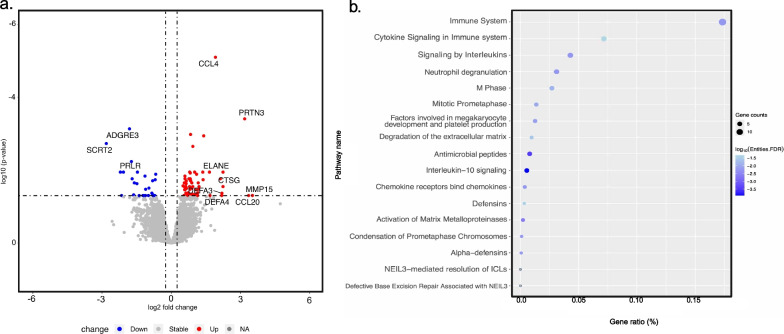
Transcriptomic assessment of latent profile phenotypes of pediatric septic shock. **a** Volcano plot showing differentially expressed genes among patients belonging to *Phenotype 1* relative to those *Phenotype 2* using a log2(fold change) threshold of ± 0.25. Overexpressed genes are shown in red. Underexpressed genes are shown in blue. The top 10 most differentially expressed genes are labeled including matrix metallopeptidase-15 (*MMP15),* chemokine ligand 20 (*CCL20*)*,* proteinase 3 (*PRTN3),* neutrophil expressed elastase (*ELANE*)*,* cathepsin G (*CTSG*)*,* defensin 3 (*DEFA3),* defensin 4 (*DEFA4*)*,* chemokine ligand 4 *(CCL4*), scratch family transcriptional repressor 2 *(SCRT2),* and adhesion G protein-coupled receptor E3 (*ADGRE3)*. **b** Biologically enriched pathways among patients with *Phenotype 1* relative to those in *Phenotype 2*. The y-axis represents the REACTOME pathways enriched for the significantly overexpressed genes. The x-axis represents the gene-ratio (%). The size of the circle indicates gene counts. The darker hue of color indicates a lower adj. *p* value

Transcriptomic data was available in 145 patients. We identified 91 differentially expressed genes (DEGs) when comparing patients with *Phenotype 1* (n = 18) versus *Phenotype 2* (n = 127), of which 62 genes were overexpressed and 29 were underexpressed. The top ten overexpressed genes with an FDR adjusted *p* value < 0.05 were *PRTN3, ELANE, CTSG, DEFA3, DEFA4, CCL4, HBB, G0S2, NEIL3,* and *CEP55*. The top ten under-expressed genes were *SCRT2, PRLR, ADGRE3, FSTL4, LGALSL, HCAR2, RAMP3, OLIG2, SHE, and CMTM2.* Biological pathways enriched among patients with *Phenotype 1* relative to those *Phenotype 2* corresponded to activation of the immune system, cytokine signaling, neutrophil degranulation, and antimicrobial peptides. CIBERSORT analyses identified that only the proportion of neutrophils was lower among patients with *Phenotype 1* relative to *Phenotype 2*. The volcano plot and results of biological pathway analyses are shown in Fig. 3.

**Fig. 4:**
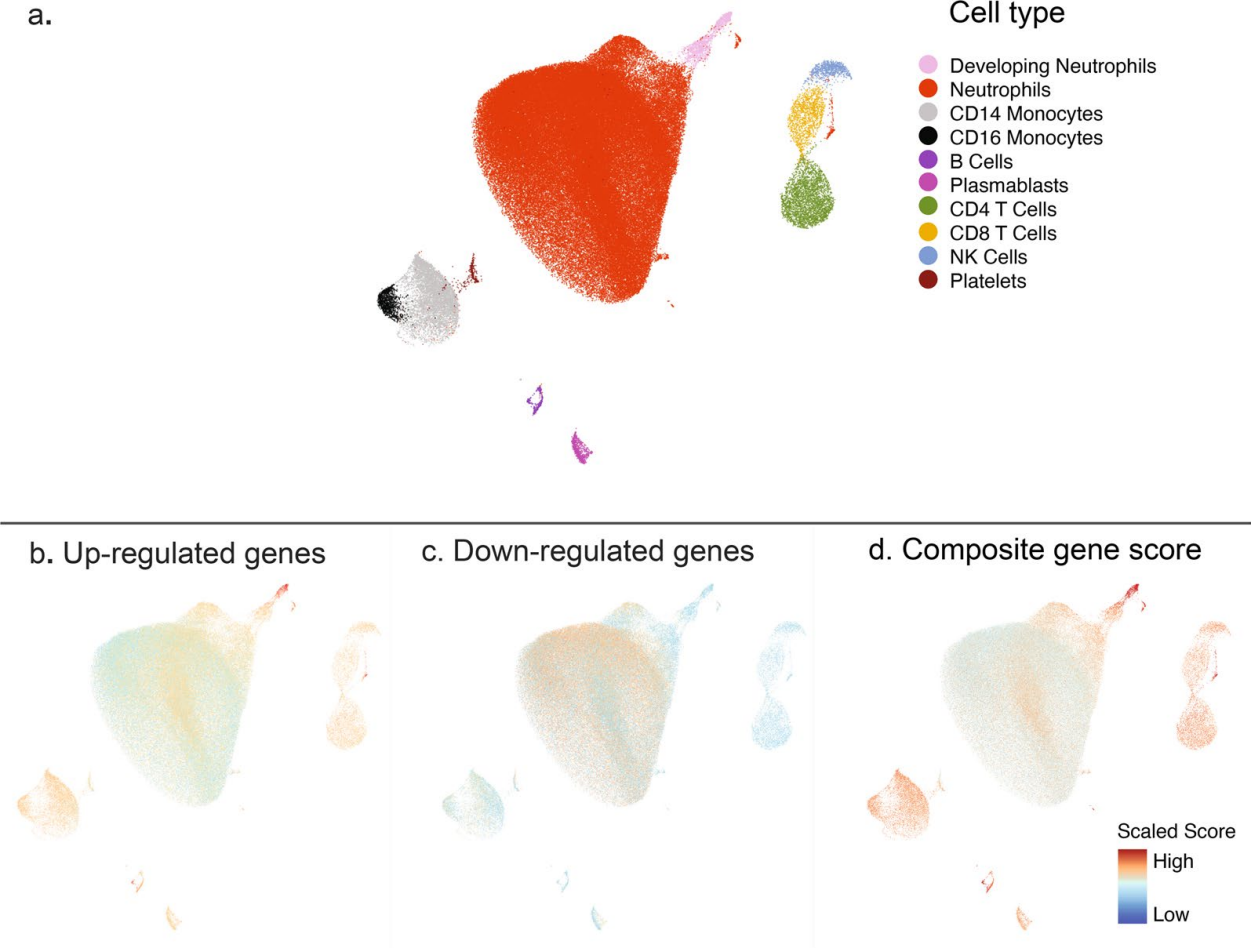
Inference of cell subsets underlying latent profile phenotypes identified in the study. The figure shows the Uniform Manifold Approximation and Projection (UMAP) of the publicly available single-cell transcriptomic dataset from critically ill adults with sepsis published by *Kwok* et al. **a** Ten cell subsets were identified in the single-cell dataset. (1) Developing neutrophils (pink), (2) Mature neutrophils (red), (3) Cluster differentiation (CD) 14 positive monocytes (light gray), (4) CD16 positive monocytes (black), (5) B lymphocytes (deep purple), (6) PB: Plasmablasts (purple), (7) CD4 positive T lymphocytes (moss green), (8) CD8 positive T lymphocytes (yellow), (9) NK: Natural killer cells (blue), and (10) Platelets (brown). **b** Upregulated genes among patients with *Phenotype 1* shown in red, **c** downregulated genes among patients with *Phenotype 1* shown in red, and **d** composite gene score representing geometric mean of upregulated minus downregulated genes among patients belonging to *Phenotype 1*. The gene score was scaled as shown in the legend. Cells in red represent those with a high composite gene score indicating that they contributed predominantly to over-expressed genes among patients with *Phenotype 1*. In contrast, cells in blue represent those with a low composite gene score indicating that they contributed predominantly to genes underexpressed among patients with *Phenotype 2*

As shown in Fig. 4, the *Kwok* et al. [26] single-cell RNAseq dataset had 10 cell types from critically ill adult patients with sepsis. Expression data of 58 over-expressed and 19 under-expressed genes identified through DEG analyses distinguishing latent profile phenotypes were available in the single-cell dataset and detailed in the Online Supplement. Genes upregulated among patients with *Phenotype 1* were expressed primarily by a small population of developing neutrophils, and to a lesser extent by CD14 and CD16 positive monocytes, CD4 and CD8 T-cells, natural killer (NK cells), and plasmablasts. Downregulated genes among patients with *Phenotype 1* were expressed primarily by mature neutrophils.

**Fig. 5:**
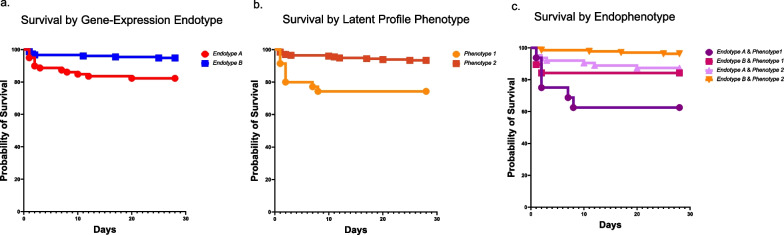
From left to right, Kaplan Meier survival curves based on **a** established gene-expression endotype (A in red vs. B in blue); Patients with *Endotype A* had a higher hazard of 28-day mortality compared to *Endotype B* (HR 3.7 (95% CI 1.5, 8.7), *p* = 0.003), **b** latent profile phenotype (*Phenotype 1* in orange and *Phenotype 2* in brown); Patients with *Phenotype 1* had a higher hazard of 28-day mortality compared to those belonging to *Phenotype 2* (HR 4.5 (95% CI 1.9, 10.6), *p* < 0.001). **c** Integrated subclass assignment scheme that considered both the endotype and phenotype assignment among patients including all four possible combinations: (i) *Endotype A*/*Phenotype 1* (deep purple), (ii) *Endotype B*/*Phenotype 1* (deep plum), (iii) *Endotype A*/*Phenotype 2* (light magenta), (iv) *Endotype B*/*Phenotype 2* (orange). Patients assigned as both *Endotype B* and *Phenotype 2* had the lowest mortality risk. Compared to this group, patients classified as *Endotype A* & *Phenotype 1* had a higher hazard of mortality (HR 12.5 (95% CI 3.8, 41.2), *p* < 0.001). Patients classified as *Endotype B* & *Phenotype 1* had a hazard ratio of mortality of 4.8 (95% CI 1.1, 20.1, *p* = 0.032). Patients classified as *Endotype A* & *Phenotype 2* had a hazard ratio of mortality of 3.6 (95% CI 1.2, 11.1), *p* = 0.024. There were no statistically significant differences between the latter two groups

A total of 233 patients in the study had data on established gene-expression endotypes and newly derived latent profile phenotype assignments. There was no statistically significant association between endotypes and phenotypes in the cohort (Pearson χ^2^ test, *p* value of 0.08). Figure 5 shows the Kaplan Meier survival curves based on gene-expression endotype (*Endotype* *A* vs. *B*), latent profile phenotype (*Phenotype 1* vs. *Phenotype 2*), and an integrated scheme where we considered all four possible combinations of endotype and phenotype assignment. Patients classified as *Endotype B* & *Phenotype 2* had the lowest mortality risk. Relative to this group, those classified as *Endotype A* & *Phenotype 1* had an over 12-fold (HR 12.5, 95% CI 3.8, 41.2, *p* < 0.001) higher hazard of mortality; those with *Endotype B* & *Phenotype 1* had a nearly fivefold higher hazard of mortality (HR; 4.8, 95% CI 1.1, 20.1, *p* = 0.032); those with *Endotype A* & *Phenotype 2* had an over threefold higher hazard of mortality (HR 3.6, 95% CI 1.2, 11.1, *p* = 0.024). There were no statistically significant differences in mortality between the latter two subclasses.

## Discussion

In this study, we derived and internally validated two pediatric septic shock phenotypes, identified through latent profile analyses, of high prognostic relevance. With one exception, there was no evidence for heterogeneous responses to common sepsis treatments on clinical outcomes between phenotypes. Transcriptomic analyses revealed overexpression of genes implicated in innate immune response among those belonging to *Phenotype 1*. Our data suggest a predominance of developing neutrophils among this high-risk subset of patients. We did not identify a statistically significant overlap between established gene-expression endotypes and the newly derived latent profile phenotypes. Finally, we demonstrated the prognostic relevance of patient endophenotypes based on an integrated subclassification scheme that considered both gene-expression-based endotypes and clinico-biomarker latent profile phenotypes.

The phenotypes identified in our study share similarities with the *hyper*- and *hypo-inflammatory* phenotypes originally described by *Calfee* and colleagues among adults with ARDS [13, 14], and subsequently reproduced among other adult [30] and pediatric patients [16]; molecular phenotypes of acute kidney injury detailed by *Bhatraju* et al. among adults [31]; and most recently those identified by Sinha et al. among septic adults [18]. Our data provide further support of the reproducibility of latent profile analyses as a methodologic approach to identify phenotypes, irrespective of assigned syndromic diagnoses, across the spectrum of the host developmental age.

We provide evidence for the prognostic utility of latent profile phenotypes with *Phenotype 1* being independently associated with significant risk of poor clinical outcomes upon adjusting for multiple potential confounders. Unlike previous studies, beyond the robust prognostic implications, we did not find evidence of HTE of common sepsis therapies on clinical outcomes among phenotypes. The exception to this was that those patients with classified as *Phenotype 1* who received ≥ 2 antimicrobial therapies had a significantly higher rate of complicated course than those belonging to *Phenotype 2* who received ≥ 2 antimicrobial therapies. While this observation may merely reflect the fact that *Phenotype 1* represented the sickest subset of patients, a few additional considerations are warranted. *Phenotype 1* may represent patients with an inadequate source control of infection, those with insufficient therapeutic drug levels of antimicrobials, and patients with an exaggerated host innate immune response, despite appropriate antimicrobial coverage. Of note, our findings mirror those of *Sinha* et al. where the authors identified that septic adults with a *hyperinflammatory* phenotype had higher rates of bacteremia than those without [18]. Pending validation, future studies are needed to determine whether precision antibiotic dosing, targeted use of extra-corporeal blood purification strategies, and or modulation of the innate immune response can be used to improve outcomes among patients with *Phenotype 1*.

We did not identify a differential response to corticosteroids among phenotypes unlike that observed among adults with COVID-19 [15]. The explanations for this difference are likely multifactorial including the relative homogeneity among patients with COVID-19 compared to the cohort studied, differences in pathogen type -viral versus bacterial induced host response, and compartmentalized effects of corticosteroids based on primary cells affected—lung versus peripheral blood. In addition, *Sinha* and colleagues demonstrate differential responses to recombinant activated protein C (rAPC) versus placebo among phenotypes when re-examining results of the PROWESS-SHOCK trial data [18]. While we demonstrate evidence of coagulopathy among those with *Phenotype 1*, we cannot comment on whether latent profile phenotypes among children would be expected to have a similar biological response as with adults, given the developmental differences in host response [32].

Transcriptomic analyses revealed activation of neutrophil pathways consistent with gene-expression studies comparing phenotypes of adult ARDS and sepsis [33–35]. Taken together CIBERSORT analyses and the single-cell composite gene-score data suggest a higher turnover of neutrophils among those with *Phenotype 1* compared to those *Phenotype 2*. These data are intuitive to the clinician and congruent with findings from single-cell multi-omics studies among septic adults, wherein patients with the worst clinical outcomes were characterized by emergency granulopoiesis and the presence of developing neutrophils [26]. Finally, our data suggest a preponderance of additional cell subsets including CD14 and CD16 monocytes, T- and NK-cells, and plasmablasts among *Phenotype 1* patients. While we cannot confidently speak to whether the phenotypes identified represent ‘treatable traits’ [36], our data indicate that the groups identified are biologically distinct. Future studies are necessary to determine the mechanistic link between cell subtypes and phenotypes, and whether targeted modulation of cell subsets can be used as a novel therapeutic approach against sepsis.

We did not identify a statistically significant overlap between established gene-expression-based endotypes and latent profile phenotypes. As such our data suggest that, fundamentally, these two approaches are sampling different, albeit vitally important, biological facets of the host response in critical illness. While the former broadly reflects the adaptive arm of the host immune response, the latter informs the innate arm of the host response, including microvascular endothelial function. Therefore, we believe that the integrated classification scheme of endophenotypes detailed in our study is of clinical and potential therapeutic relevance. For instance, patients classified as *Endotype A* & *Phenotype 1* may represent an extreme endophenotype with a significantly increased risk of mortality. This is consistent with the observation that critically ill patients with repressed adaptive- and overactive innate- immune responses have been consistently associated with the worst clinical outcomes [37]. As such these patients would be expected to be poor candidates to receive corticosteroids based on their endotype. However, they may potentially benefit from targeted interventions or immunomodulation to quell the innate immune response based on their phenotypic assignment. Furthermore, although patients with *Endotype B* & *Phenotype 1* and *Endotype A* & *Phenotype 2* endophenotypes had comparably elevated risk of mortality, the therapeutic implication of such subclass assignment is expected to be diametrically opposite between groups. Although speculative, pending validation in cohort studies and clinical trials, such an integrated subclassification scheme holds the potential to inform better alignment of interventions among those critically ill by providing a comprehensive understanding of patient-level pathobiology [38].

Our study has several limitations: (1) the observational nature of the study limits precludes inference of causality; (2) despite accounting for era of patient enrollment in our multivariate models, the long study period is a limitation; (3) data missingness especially for biomarker data is a limitation. However, this was mitigated by the use of robust imputation approaches and sensitivity analyses, the latter demonstrating unchanged associations with exclusion of imputed variables in the validation dataset. (4) latent profile phenotypes were based on day 1 data. However, given the temporal and dynamic nature of the host response, it is conceivable that these class assignments may be subject to change over time; (5) external validation dataset to demonstrate the reproducibility of our SVM model was lacking. Moreover, we did not seek to develop a classifier that used a parsimonious set of predictor variables as this is better achieved in external validation sets; (6) given the observational nature of the underlying cohort, interaction effect based upon receipt of ≥ 2 antimicrobials among phenotypes on odds of complicated course is speculative. Although we attempted to address confounding by indication by using IPTW analyses, these data should be interpreted with caution. (7) the number of patients with *Phenotype 1* among whom transcriptomic data was available was limited, which may have contributed to fewer DEGs being identified; (8) the integrated single-cell data used as reference was largely comprised of samples obtained from adults with sepsis rather than pediatric patients. Further, prospective studies that simultaneously capture phenotypic and single-cell transcriptomic data are necessary to directly identify cell subsets underlying pediatric critical illness subclasses; (9) the number of patients in whom both established gene-expression endotype and latent profile phenotype class assignments were available was limited; (10) both endotype and phenotype assignments were based on data generated within 24 h of meeting septic shock criteria and were assumed to reflect baseline differences in host response. However, a significant proportion of patients in the cohort received corticosteroids. It remains plausible that the biological differences in host response among subclasses may reflect those in response to corticosteroids, rather than baseline differences.

## Conclusions

In this study, we demonstrate the existence of two phenotypes among children with septic shock identified through latent profile analyses with high prognostic value. We provide evidence of upregulated host innate responses including microvascular endothelial dysfunction among those with *Phenotype 1* with transcriptomic evidence of high turnover of neutrophils. The phenotypes did not show overlap with established gene-expression-based endotypes in pediatric septic shock nor demonstrate a differential response to corticosteroids. We integrated these two promising classification schemes to delineate novel sepsis ‘endophenotypes’. Pending validation, such an approach may allow for therapeutic drug selection informed by a comprehensive understanding of patient-level pathobiology.

### Supplementary Information

Additional file 1.

## Data Availability

All de-identified clinical data and bulk messenger RNA sequencing (fastq) files and related metadata are available upon reasonable request to the corresponding author.
