# Supplementary material for: Identification and transcriptomic assessment of latent profile pediatric septic shock phenotypes
Source: Crit Care. 2024 Jul 17;28:246. doi: 10.1186/s13054-024-05020-z (PMC11253460; doi:10.1186/s13054-024-05020-z)
Supplement: Supplementary file 1 — Additional file 1. [file 13054_2024_5020_MOESM1_ESM.pdf]

Online Supplement

Identification and transcriptomic assessment of latent profile pediatric septic shock phenotypes.

Mihir R. Atreya, MD, MPH[1,2]; Min Huang, BS[3]; Andrew R. Moore, MD[4]; Hong Zheng, PhD[4,5]; Yehudit Hasin-Brumshtein, PhD[6]; Julie C. Fitzgerald, MD, PhD[7]; Scott L. Weiss, MD[8]; Natalie Z. Cvijanovich, MD[9]; Michael T. Bigham, MD[10]; Parag N. Jain, MD[11]; Adam J. Schwarz, MD[12]; Riad Lutfi, MD[13]; Jeffrey Nowak, MD[14]; Neal J. Thomas, MD[15]; Michael Quasney, MD[16]; Mary K. Dahmer, PhD[16]; Torrey Baines, MD[17;] Bereketeab Haileselassie, MD[18]; Andrew J. Lautz, MD[1,2]; Natalja L. Stanski, MD[1,2]; Stephen W. Standage, MD[1,2]; Jennifer M. Kaplan, MD, MS,[1,2]; Basilia Zingarelli, MD, PhD[1,2]; Rashmi Sahay, MS[19]; Bin Zhang, PhD[19]; Timothy E. Sweeney, MD, PhD[6;] Purvesh Khatri, PhD[4,5]; L. Nelson Sanchez-Pinto, MD, MBI[20,21]; and Rishikesan Kamaleswaran, PhD.[22,23]

Author Affiliations:

1. Division of Critical Care Medicine, Cincinnati Children's Hospital Medical Center, Cincinnati, OH, 45229, USA.
2. Department of Pediatrics, University of Cincinnati College of Medicine, Cincinnati, OH, 45627, USA.
3. Department of Biomedical Informatics, Emory University School of Medicine, Atlanta, GA, USA.
4. Stanford Institute for Immunity, Transplantation and Infection, Stanford University School of Medicine, Stanford, CA,
5. Center for Biomedical Informatics Research, Department of Medicine, Stanford University School of Medicine, Stanford, 94305, CA.
6. Inflammatix, Sunnyvale, CA, 94085, USA.
7. Children's Hospital of Philadelphia, Philadelphia, PA, 19104, USA.
8. Nemours Children's Health, Wilmington, DE, 19803, USA.
9. UCSF Benioff Children's Hospital Oakland, Oakland, CA, 94609, USA.
10. Akron Children's Hospital, Akron, OH, 44308, USA.
11. Texas Children's Hospital, Baylor College of Medicine, Houston, TX, 77030, USA.
12. Children's Hospital of Orange County, Orange, CA, 92868, USA.
13. Riley Hospital for Children, Indianapolis, IN, 46202, USA.
14. Children's Hospital and Clinics of Minnesota, Minneapolis, MN, 55404, USA.
15. Penn State Hershey Children's Hospital, Hershey, PA, 17033, USA.
16. C.S Mott Children's Hospital, University of Michigan, Ann Arbor, MI, 48109, USA.
17. University of Florida Health Children's Hospital, Gainesville, FL, 32610, USA.
18. Lucile Packard Children's Hospital Stanford, Palo Alto, CA, 94304, USA.
19. Division of Biostatistics and Epidemiology, Cincinnati Children's Hospital Medical Center Cincinnati, 45229, OH, USA.
20. Department of Pediatrics, Northwestern University Feinberg School of Medicine, Chicago, 60611, IL, USA.
21. Department of Health and Biomedical Informatics, Northwestern University Feinberg School of Medicine, Chicago, 60611, IL, USA.
22. Department of Biomedical Informatics, Emory University School of Medicine, Atlanta, 30322, GA, USA.

23. Department of Biomedical Engineering, Georgia Institute of Technology, Atlanta, 30322, GA, USA.

Corresponding author:
Mihir R Atreya, MD, MPH
Cincinnati Children's Hospital Medical Center
Division of Critical Care Medicine, MLC2005
3333 Burnet Avenue
Cincinnati, OH, 45229, USA
Tel: 513-636-1627
Email: Mihir.Atreya@cchmc.org

Online Supplement

Index:

1. Supplementary Methods……………………………………………………………4
2. Supplementary Tables…………………………………………………………..11
3. Supplementary Figure Legend…………………………………………………18
4. Supplementary Figures………………………………………………….................21
5. References…………………………………………………………………………32

Online Supplement

Supplementary methods:

Study design and patient selection: The study protocol was approved by Institutional Review Boards (IRBs) of the primary site (Cincinnati Children's Hospital IRB, Study Title: 'Genomic Analysis of Pediatric Systemic Inflammatory Syndrome', IRB ID: 2008-0558, Initial Approval 5/9/2002, Most Recent Approval: 6/22/2023) as well as the 13 participating institutions. Informed consent was obtained from parents or guardians of patients.

Inputs for derivation of phenotypes: Vital sign data (heart rate, respiratory rate, systolic-, diastolic-, and mean arterial- pressure) collected within 24 hours of meeting study criteria were available in all patients. Given the significant variability in vital signs across the pediatric age spectrum, we measured deviation of patient vital sign from the median for age and sex for heart- and respiratory- rates, (1) systolic-, diastolic-, and mean arterial- blood pressures. (2) We calculated peak vasoactive inotropic score (VIS) on day 1.(3) Laboratory data were collected at the discretion of treating physicians including arterial or venous blood gases: pH, pCO2 (mmHg), and base excess (BE); serum lactate, sodium (Na), potassium (K), chloride (Cl), bicarbonate (HCO3), glucose (Gluc), blood urea nitrogen (BUN), creatinine (Cr), serum glutamic pyruvic transaminase (SGPT), serum glutamic-oxaloacetic transaminase (SGOT), hematocrit (HCt), platelet count, international normalized ratio (INR), all measured on day 1. Biomarker data were previously measured using multiplex Luminex assays (R&D Biosystems) in serum specimens collected on day 1 and included interleukin-8 (IL-8), intercellular adhesion molecule-1 (ICAM-1), soluble thrombomodulin (sTM), angiopoietin-1 (Angpt-1), angiopoietin-2 (Angpt-2), and tyrosine kinase with immunoglobulin-like loops and

epidermal growth factor homology domains-2 (Tie-2). (4–7) We calculated Angpt-2/Angpt-1 and Angpt-2/Tie-2 ratios. Biomarkers were $\log_2$ transformed prior to analyses to reduce skewness.

Data cleaning: We excluded variables with ≥40% missingness of data. Among those with <40% missingness, we used python package "Datawig", that uses deep learning feature extraction with automatic hyperparameter tuning to impute missing values.(8) The percent of missingness for each variable included in modeling is detailed in **Table E1.** We measured Pearsons' correlation coefficients between the clinical, laboratory, and biomarker data included in the study, presented in **Figure E1**. As shown, systolic, diastolic, and mean arterial blood pressures were positively correlated ($\rho>0.5$). However, given that these variables are independently clinically relevant in the context of vasoactive dependent septic shock patients, we chose to include all of them for further analyses. Similarly, Angpt-2, Angpt-1, and Tie-2 along with Angpt-2/Angpt-1 and Angpt-2/Tie-2 ratio demonstrated significant correlations. Once again, we chose to include each of them as their implications for biology may be different when considering individual biomarkers alone vs. ratios of biomarkers.

Random assortment of cohort into derivation and validation datasets: We generated and assigned random numbers for all participants included in the dataset. Subsequently, we sorted the patients based on the random number from smallest to largest. The first 60% of the cohort were included in the derivation dataset and the remaining 40% were included in the validation dataset.

Latent profile analysis (LPA) is a Gaussian Finite Mixture Modeling method that attempts to identify clusters of individuals based on responses to a series of continuous

variables. It assumes that there are unobserved latent profiles that generate patterns of responses on indicator items. Unlike latent class analyses (LCA), LPA only uses continuous variables. LPA is a probabilistic model, which means that it models the probability of a case belonging to a profile. This is thought to be a superior approach such as *K-means* clustering, which uses distance algorithms. The R package *mclust* performs various types of model-based clustering and dimensionality reduction. It requires complete data.

Selection of optimal model: Bayesian Information Criteria (BIC) was used to select the optimal number of latent profiles in the derivation set. BIC for all models is shown in **Figure E2**. The output describes the geometric characteristics of the profiles. The *summary (BIC)* function in *mclust* identified the top three models, shown in **Table E2**. We used additional criteria including the Integrated Complete-data Likelihood (ICL) criterion, which penalizes solutions with greater entropy or classification uncertainty. The ICL for all models are shown in **Figure E3.** The *summary (ICL)* function identified the top three models, shown in **Table E3.** Additionally, we generated LPA plots of models with 3, 4, and 5 profiles, shown in **Figures E4-6**. The difference between groups was less clear with an increasing number of profiles. Thus, we chose the two-profile model presented in the manuscript.

Support vector machine (SVM) classifier: Using the phenotypic assignment in the derivation set, we trained a SVM classifier to assign phenotypes in the hold-out validation set. We used python program “scikit-learn” to develop a non-probabilistic binary linear classifier to distinguish phenotypes. Briefly, grid search was used to obtain optimal parameters including linear kernel and a regularization parameter C of 0.1. Of

note, the SVM classifier developed utilized the same clinical, laboratory and biomarker variables included in the LPA model in the derivation cohort to assign phenotypes in the internal validation set. The comparison between demographic, patient characteristics, and outcomes between phenotypes in the derivation and validation sets, is presented in **Table 1** of the main manuscript, which demonstrates the reproducibility of our SVM classifier. Comparison of key clinical, laboratory, and biomarkers distinguishing phenotypes in the validation set with exclusion of imputed data shown in **Figure E7.** These data show that patient phenotypes are biologically distinct. Furthermore, a subset of patients in the cohort had biomarker data on interleukin-6 (IL-6) and soluble tumor necrosis factor receptor (sTNFr)**.** While we did not include these data in the derivation of phenotypes due to high missingness, we compared differences in biomarkers between phenotypes. Consistent with latent class phenotypes among several studies of critical illness, these biomarkers were significantly higher among patients with *Phenotype 1* compared to those classified as *Phenotype 2*. These data are shown in **Figure E8.**

Transcriptomic analyses: Bulk messenger RNA sequencing data were available from a subset of the cohort recruited between 2019 and 2023 with RNA processed from whole blood collected in PAXgene® tubes on day 1 of septic shock. We used ComBat_seq function from the sva package (v.3.46.0), a negative binomial regression method, to eliminate batch effects in the transcriptomic data. We used DESeq2 (v.1.38.3) to identify differentially expressed genes (DEGs) between the phenotypes identified across derivation and validation sets. DEGs were selected based on ≥log2 fold change value cutoff of ±0.25, and adjusted p value of 0.05. We conducted Reactome pathway analyses with a Benjamin Hochberg false discovery rate (FDR) <

0.05 to identify enriched biological pathways and CIBERSORT analyses, a bulk deconvolution approach, to determine differences in cell subsets between phenotypes, the latter detailed in **Figure E9** of the online supplement.

Inference of cell subsets using publicly available single-cell data:

Deconvolution approaches have several important limitations including the fact that most algorithms are based on differences in cell types in tissue homogenates rather than whole blood and often lack data on cell specific markers in disease states. Thus, we sought to gain a granular insight at a single-cell level into immune cell subpopulations associated with latent profile phenotypes. To achieve this, we used single-cell RNA sequencing dataset comprised of critically ill adults with sepsis published by *Kwok* et al.(9)**.** Briefly, gene expression-data from this dataset was explored using the Seurat (v4.0.5), and cells were analyzed by Uniform Manifold Approximation and Projection (UMAP) and nearest neighbors' algorithm demonstrating distinct cell types. We evaluated the cellular origin of differentially expressed genes (DEGs) between latent profile phenotypes identified in our study by referencing expression of genes against the single-cell dataset. Overexpressed (58 out of 62 up DEGs) and under-expressed genes (19 out of 29 DEGs) which were also available in the *Kwok* dataset are shown in **Figure E10** and **11**. A composite gene score was calculated as the geometric mean of available overexpressed genes minus the geometric mean of under-expressed genes and mapped against the single cell level using UMAP in **Figure 4** of the manuscript, according to published methods.(10)

Heterogeneity of treatment effect: We used inverse probability treatment weighting (IPTW) to test the effect of common sepsis treatments on the odds 28-day mortality -a

secondary outcome- among latent profile phenotypes accounting for the effect of multiple confounding variables. Treatments tested included use of >100 ml/kg vs. < 100 ml/kg fluid resuscitation, ≥ 2 vs. < 2 antimicrobials, ≥ 2 vs. < 2 vasoactive medications on day 1, and corticosteroid use. For IPTW models, we adjusted for age, PRISM-III score, day 1 vasoactive inotropic score (VIS), presence of comorbidity and immunocompromised status. Interaction p-values for overall effect were used to test for heterogeneity of treatment effect (HTE) across latent profile phenotypes on 28-day mortality. Unadjusted, inverse probability treatment weighting (IPTW) adjusted association, and overall interaction between latent profile phenotypes and common sepsis treatments on odds of 28-day mortality in the cohort is shown in **Table E4.**

Established gene-expression endotypes: Using a customized multiplexed panel of 100 genes, reflective of the adaptive immune response and glucocorticoid receptor signaling, mRNA expression was directly quantified by the NanoString nCounter platform. (11) The panel included 4 housekeeping genes including b-2-microglobulin, folylpolyglutamate synthase, 2,4-dienoyl coenzyme A reductase 1, and peptidylprolyl isomerase B. Image analysis of gene-expression mosaics generated using gene-expression dynamics investigator (GEDI) were previously used to assign pediatric septic shock *endotypes A* and *B* in a subset of the cohort.(11)

Overlap between these established gene-expression endotypes and newly derived latent profile phenotypes are shown in **Table E5.** The number-at-risk in Kaplan Meier Survival Curves detailed in **Figure 5** of the manuscript according to established gene-expression endotype, latent profile phenotype, and integrated endophenotype subclassification scheme is shown in **Table E6**. The Cox proportional hazard of 28-day

mortality comparing ‘endophenotypes’ based on Cox regression analyses relative to reference group with the lowest mortality is shown in **Table E7.**

Online Supplement

Table E1. Percent of missing data for clinical, laboratory, and biomarker data variables across the derivation and validation phases of the study.

Variable	Percent Missing
Age	0.0
VIS	0.0
Temp	0.0
Heart rate	0.0
Respiratory rate	0.0
Systolic blood pressure	0.0
Diastolic blood pressure	0.0
Mean arterial pressure	0.0
Sodium (Na)	1.7
Potassium (K)	1.7
Chloride (Cl)	1.8
Bicarbonate (HCO3)	1.8
Blood urea nitrogen (BUN)	2.1
Creatinine (Cr)	2.1
Glucose (Gluc)	3.4
pH	4.4
Hct	4.6
PCO2	4.9
Platelet	5.2
WBC	5.7
BE	8.2
Lactate	21.8
SGPT	36.9
ICAM-1	37.1
sTM	37.1
Angpt2	37.1
Tie2	37.1
Angpt2/Tie2	37.1
SGOT	37.2
Angpt1	37.6
Angpt2/Angpt1	37.6
IL-8	37.7
INR	39.7

Table E2.

The top 3 models and number of latent profiles based on maximum Bayesian Information Criteria (BIC), ordered from best model from left to right, in the derivation set.

	VEV, 2	VEI, 5	EEV, 2
**BIC**	-127603.7	-129076.5	-1290270.9
**BIC diff**	0.0	-1472.72	-1667.124

VEV: ellipsoidal, equal shape.

VEI: diagonal, varying volume, equal shape.

EEV: ellipsoidal, equal volume, and equal shape.

The number of optimal profiles is indicated next to each model.

Table E3.

The top 3 models and number of latent profiles based on maximum Integrated Completed Likelihood (ICL) criterion, ordered from best model from left to right, in the derivation set.

	**VEV, 2**	**VEI, 9**	**VEI, 8**
**ICL**	-127607.0	-128215.3	-128334.7
**ICL diff**	0	-608.2161	-727.6905

VEV: ellipsoidal, equal shape.

VEI: VEI: diagonal, varying volume, equal shape.

The number of optimal profiles is indicated next to each model.

Online Supplement

Table E4. Unadjusted, inverse probability treatment weighting (IPTW) adjusted association, and overall interaction between latent profile phenotypes and common sepsis treatments on odds of 28-day mortality in the cohort.

Treatment effect	***Phenotype 1***		***Phenotype 2***		
	OR (95% CI)	P-value	OR (95% CI)	P-value	P interaction
**>100 ml/kg fluid**					
Unadjusted	1.88 (1.00 - 3.56)	0.052	1.17 (0.72 - 1.89)	0.5364	0.175
IPTW Adjusted	1.36 (0.91-2.03)	0.1323	1.25 (0.89 - 1.74)	0.1952	
**≥ 2 Antimicrobials**					
Unadjusted	1.58 (0.55 - 4.55)	0.3988	0.74 (0.35 - 1.54)	0.4189	0.2482
IPTW Adjusted	1.4 (0.91 - 2.15)	0.1307	0.62 (0.45 - 0.84)	0.0022	
**≥ 2 Vasoactives**					
Unadjusted	1.85 (1.00 - 3.43)	0.0497	1.52 (0.95 - 2.43)	0.0832	0.5405
IPTW Adjusted	2.15 (1.38 - 3.34)	0.0007	1.3 (0.92 - 1.81)	0.1338	
**Corticosteroids use**					
Unadjusted	2.15 (1.08 - 4.28)	0.0296	2.85 (1.67 - 4.88)	0.0001	0.476
IPTW Adjusted	1.38 (0.91 - 2.1)	0.134	2.17 (1.53 - 3.07)	<.0001	

*Inverse probability treatment weighting (IPTW) models adjusted for age, PRISM-III score, vasoactive inotropic score (VIS), co-morbidity, and immunocompromised status.

Table E5:

Table showing overlap of established gene-expression pediatric septic shock endotypes and latent profile phenotypes in a subset of the cohort.

	**Inflammed (n=35)**	**Uninflammed (n=198)**	**X^2 p-value**
**Endotype A** (n=79)	16	63	0.079
**Endotype B** (n=154)	19	135	

Table E6:

Number-at-risk in Kaplan Meier Survival Curves by established gene-expression endotype, latent profile phenotype, and integrated endophenotype subclassification scheme.

Study day	0	7	14	21	28
**Number-at-risk by gene-expression endotype**					
Endotype A	79	70	67	66	65
Endotype B	154	152	152	148	146
**Number-at-risk by latent profile phenotype**					
Phenotype 1	35	28	27	27	26
Phenotype 2	198	192	192	187	185
**Number-at-risk by endophenotype**					
Endotype A & Phenotype 1	16	12	11	11	10
Endotype B & Phenotype 1	19	17	17	17	16
Phenotype 2	63	59	57	56	55
Endotype B & Phenotype 2	135	135	133	132	130

Table E7:

Cox proportional hazard of 28-day mortality comparing endophenotypes relative to reference group with the lowest mortality.

**Subclass**	**Cox proportional hazard**	**P value**
Endotype A & Phenotype 1	12.5 (3.8, 41.2)	<0.0001
Endotype B & Phenotype 1	4.8 (1.2, 20.1)	0.032
Endotype A & Phenotype 2	3.6 (1.2, 11.1)	0.024

*Relative to *Endotype B/Phenotype 2*.

Online Supplement

Supplementary Figure Legend:

Figure E1: Correlation matrix for clinical, laboratory, and biomarker variables in the dataset. The Pearson's correlation coefficient is shown with positive correlation shown in blue and negative correlation represented in red.

Figure E2. Bayesian information criteria (BIC) for selection of number of latent profile phenotypes. The y-axis shows the BIC with high numbers at the top and lower numbers at the bottom. The x-axis shows change in BIC with increasing number of profiles from 1 to 9. The 14 models are named based on the geometric characteristics of the profiles. Legend: (a) "EII" spherical, equal volume, (b) "VII" spherical, unequal volume, (c) "EEI" diagonal, equal volume and shape, (d) "VEI" diagonal, varying volume, equal shape, (e) "EVI" diagonal, equal volume, varying shape, (f) "VVI" diagonal, varying volume and shape, (g) "EEE" ellipsoidal, equal volume, shape, and orientation, (h) "EVE" ellipsoidal, equal volume and orientation, (i) "VEE" ellipsoidal, equal shape and orientation (*), (j) "VVE" ellipsoidal, equal orientation, (k) "EEV" ellipsoidal, equal volume and equal shape, (l) "VEV" ellipsoidal, equal shape, (m) "EVV" ellipsoidal, equal volume, (n) "VVV" ellipsoidal, varying volume, shape, and orientation. The VEV model with 2 profiles had the highest BIC and was chosen for derivation of phenotypes.

Figure E3. Integrated Completed Likelihood (ICL) for selection of number of latent profile phenotypes. The y-axis shows the ICL with high numbers at the top and lower numbers at the bottom. The x-axis shows change in ICL with increasing number of profiles from 1 to 9. The 14 models are named based on the geometric characteristics of the profiles. Legend: (a) "EII" spherical, equal volume, (b) "VII" spherical, unequal volume, (c) "EEI" diagonal, equal volume and shape, (d) "VEI" diagonal, varying

volume, equal shape, (e) "EVI" diagonal, equal volume, varying shape, (f) "VVI" diagonal, varying volume and shape, (g) "EEE" ellipsoidal, equal volume, shape, and orientation, (h) "EVE" ellipsoidal, equal volume and orientation, (i) "VEE" ellipsoidal, equal shape and orientation (*), (j) "VVE" ellipsoidal, equal orientation, (k) "EEV" ellipsoidal, equal volume and equal shape, (l) "VEV" ellipsoidal, equal shape, (m) "EVV" ellipsoidal, equal volume, (n) "VVV" ellipsoidal, varying volume, shape, and orientation. The VEV model with 2 profiles had the highest ICL and was chosen for derivation of phenotypes.

Figure E4. Standardized mean (z-scores) for continuous class predicting variables in the derivation set based on three latent profiles.

Figure E5. Standardized mean (z-scores) for continuous class predicting variables in the derivation set based on four latent profiles.

Figure E6. Standardized mean (z-scores) for continuous class predicting variables in the derivation set based on five latent profiles.

Figure E7. Differences in clinical, laboratory, and biomarker data between latent profile phenotypes in the validation set after exclusion of imputed data demonstrate that the phenotypes retained biological distinctiveness.

Figure E8. Differences in interleukin-6 (IL-6) and soluble tumor necrosis factor receptor 4 (sTNFR-1) among phenotypes across derivation and validation sets.

Figure E8. Results of CIBERSORT deconvolution analyses. The y-axis represents the proportion of cells attributed to each cell type among patients with *Phenotype 1* (shown in orange) and *Phenotype 2* (shown in brown) phenotype. The x-axis shows the various

cell types as determined by CIBERSORT. The proportion of mature neutrophils was found to be statistically significantly lower among patients with belonging to *Phenotype 1* phenotype as compared to those with *Phenotype 2.*

Figure E10: List of overexpressed genes among patients with *Phenotype 1* referenced against the Kwok et al. single cell dataset and projected using Uniform Manifold Approximation and Projection (UMAP).

Figure E11: List of under-expressed genes among patients belonging to *Phenotype 1* referenced against the Kwok et al. single cell dataset and projected using Uniform Manifold Approximation and Projection (UMAP).

Online Supplement

Figure E1:

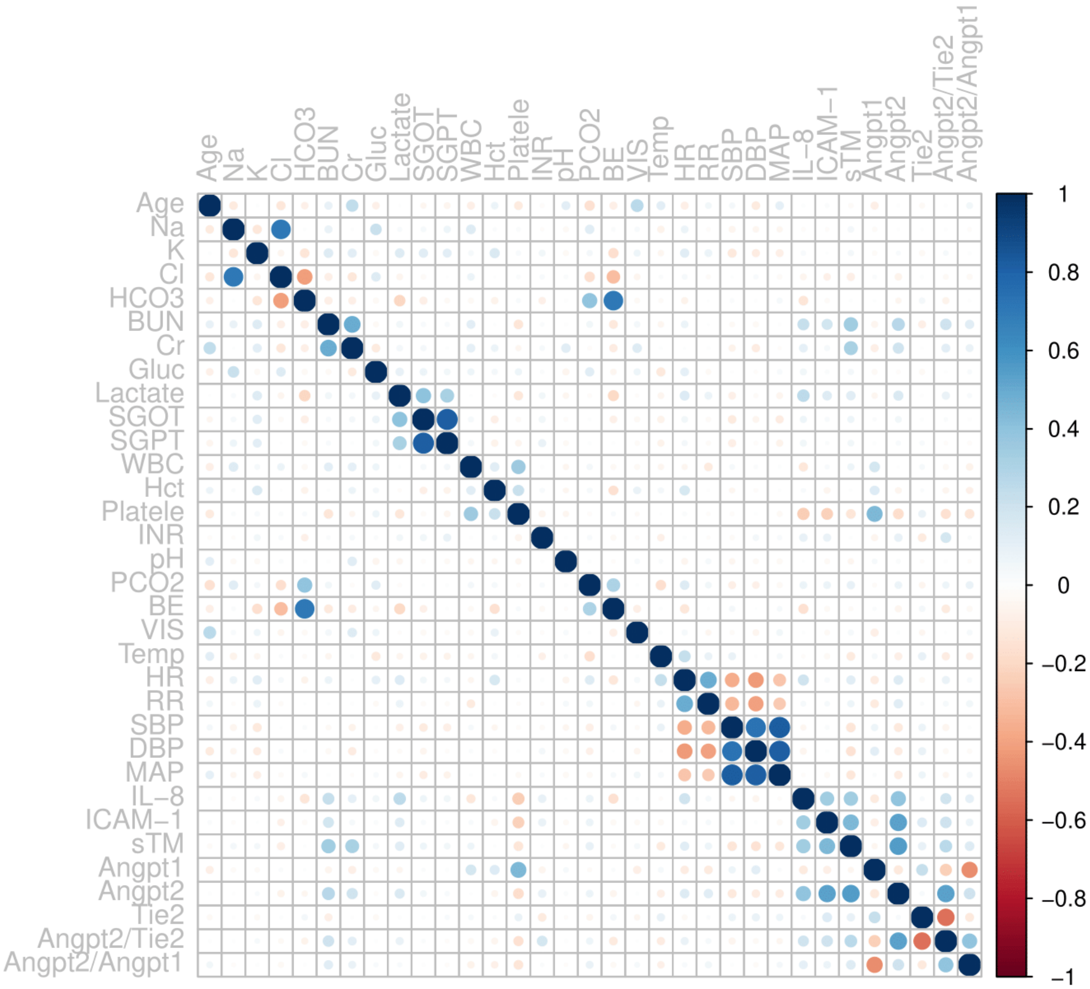

Online Supplement

Figure E2.

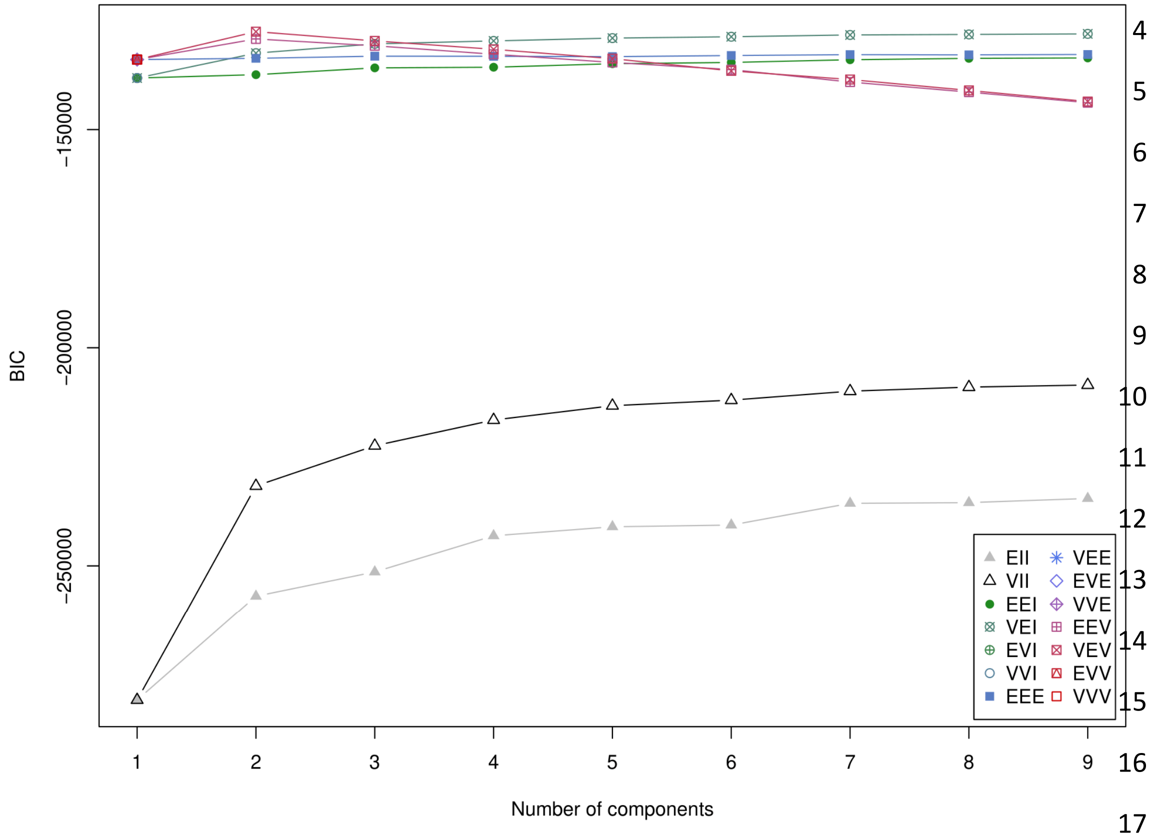

Figure E3.

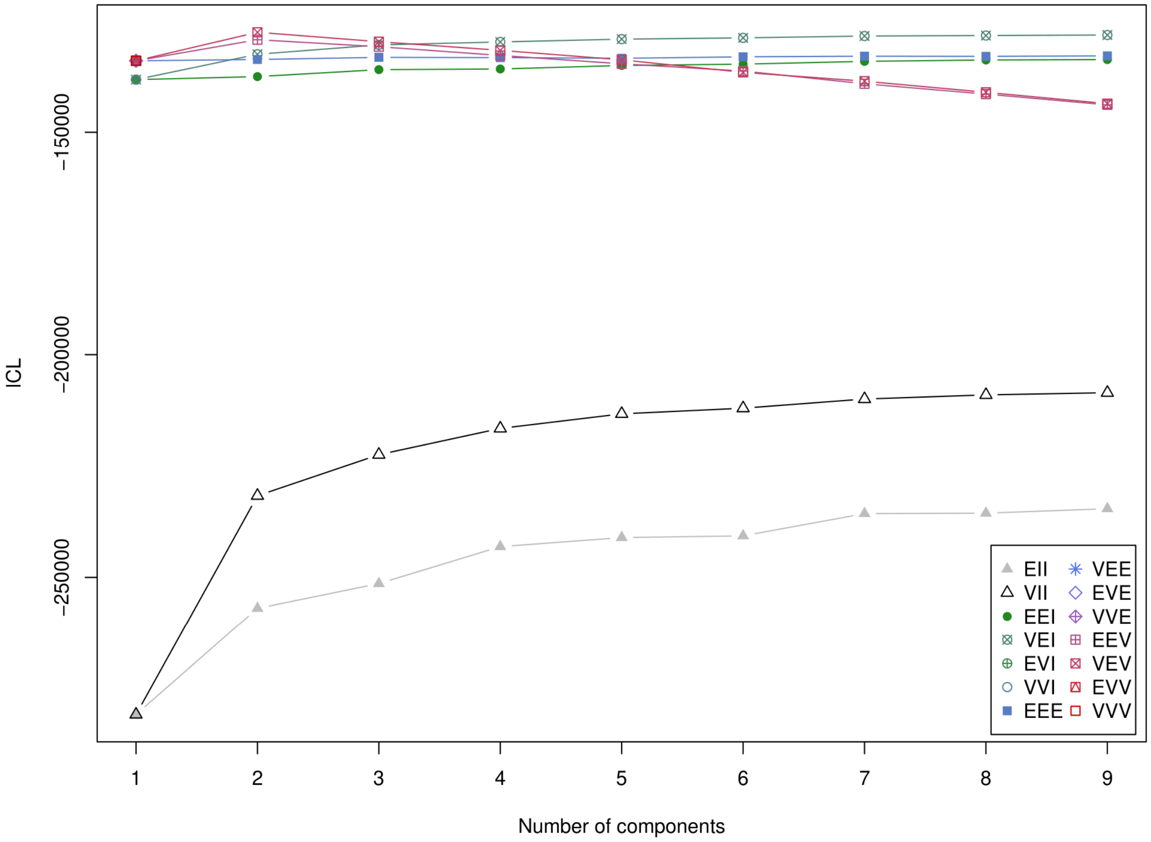
ICL
−150000
−200000
−250000
1
2
3
4
5
6
7
8
9
Number of components
EII
VII
EEI
VEI
EVI
VVI
EEE
VEE
EVE
VVE
EEV
VEV
EVV
VVV

Online Supplement

Figure E4.

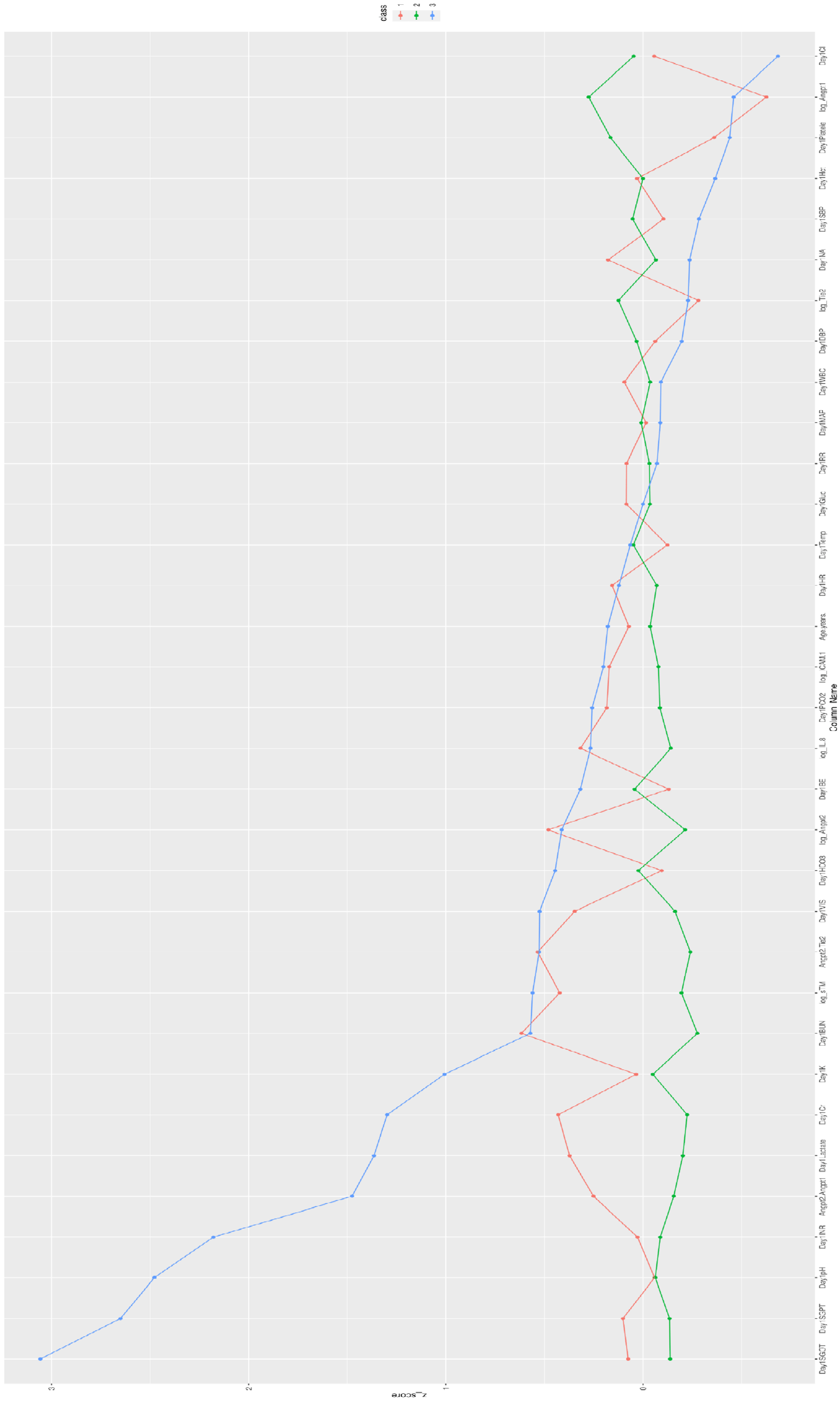

Online Supplement

Figure E5.

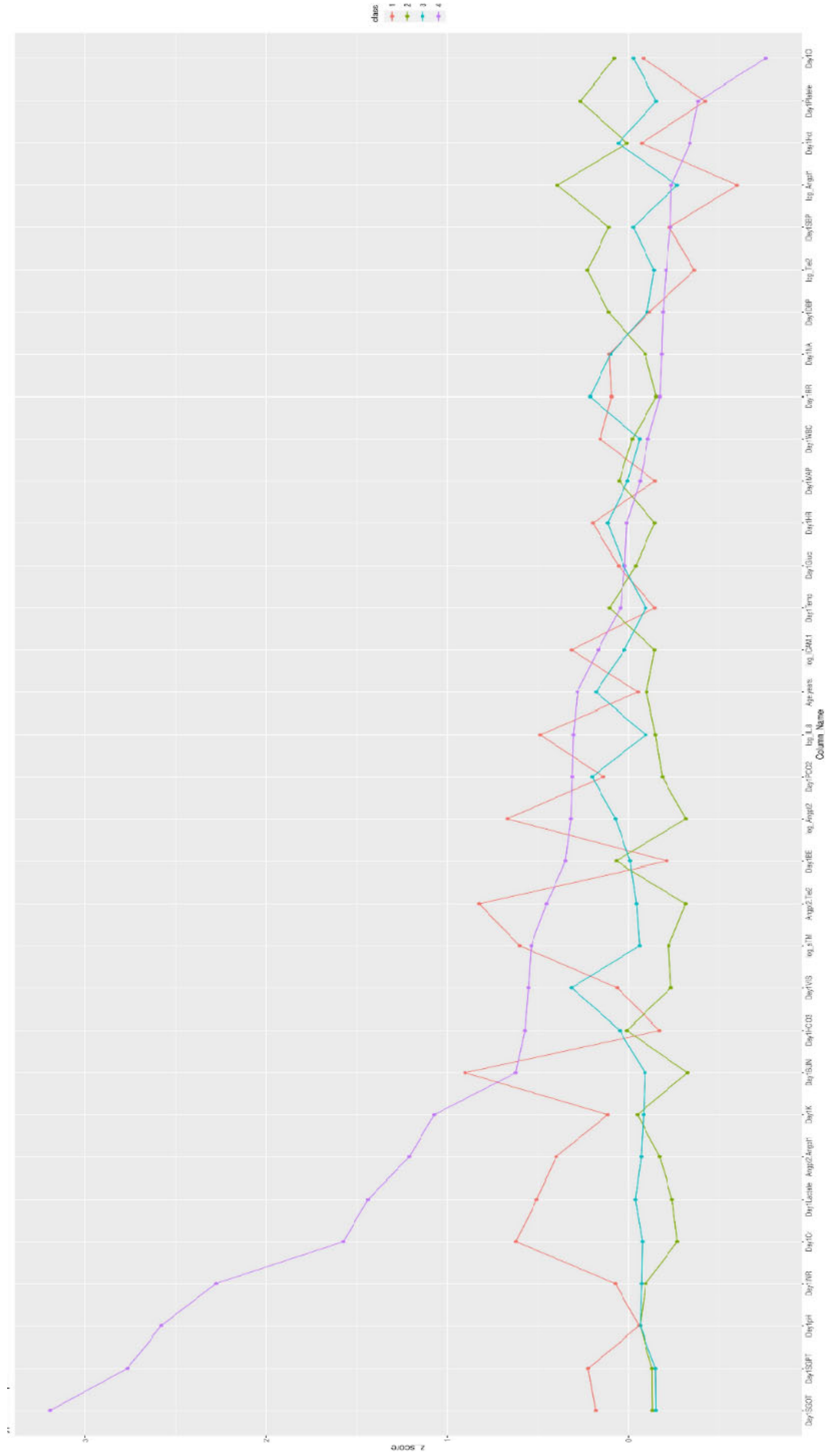

Online Supplement

Figure E6.

.

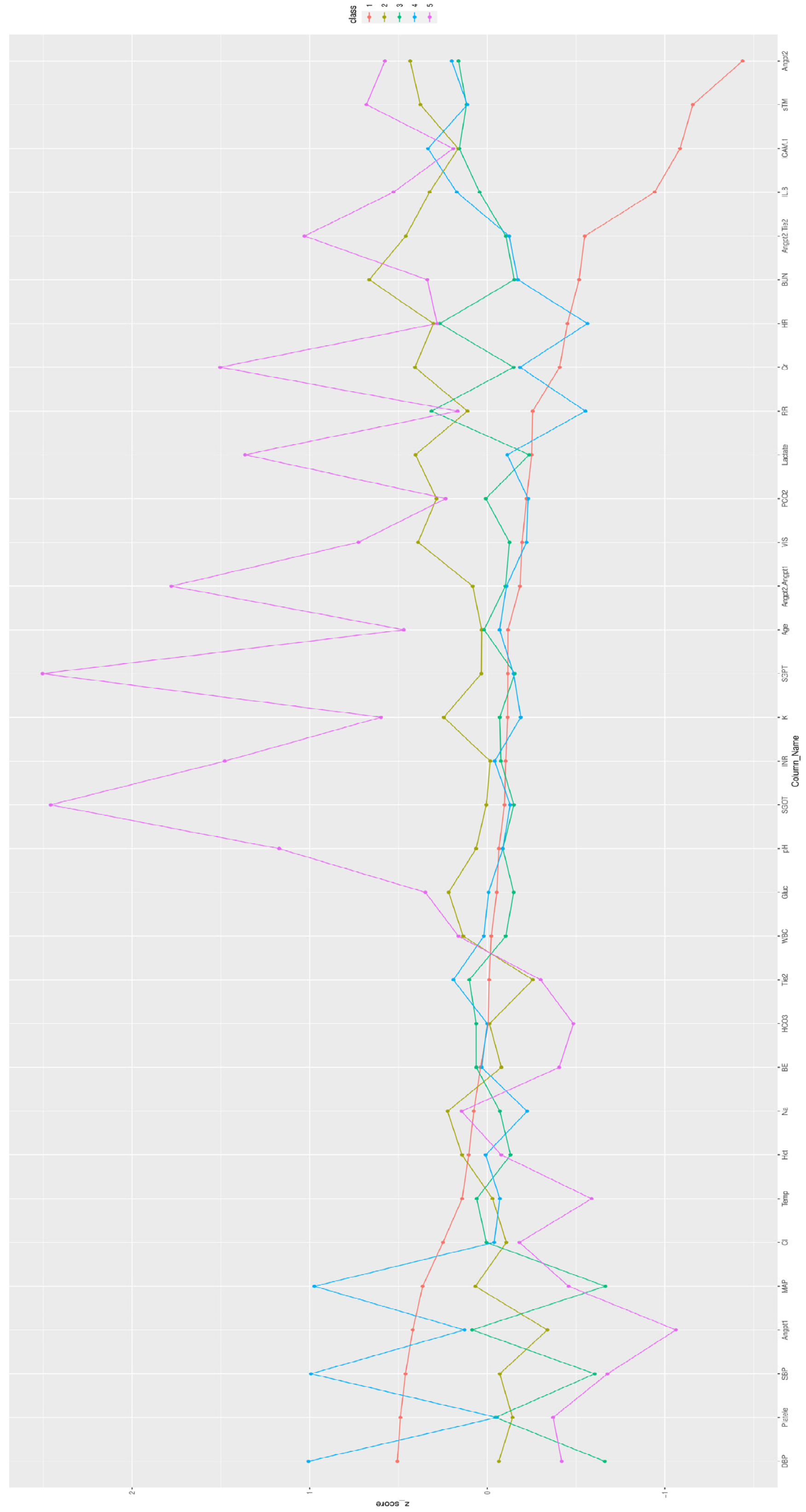

Online Supplement

Figure E7.

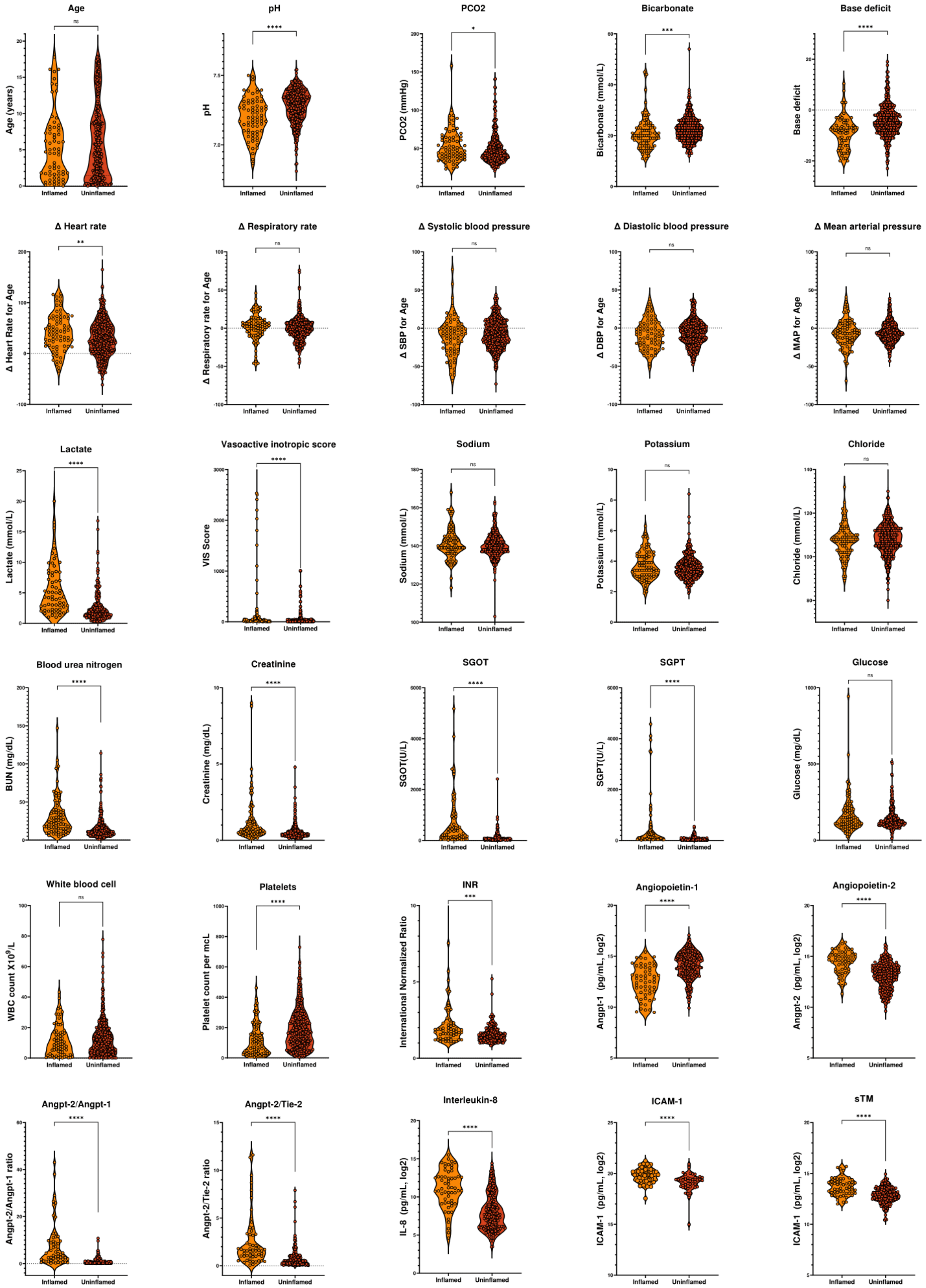

Figure E8.

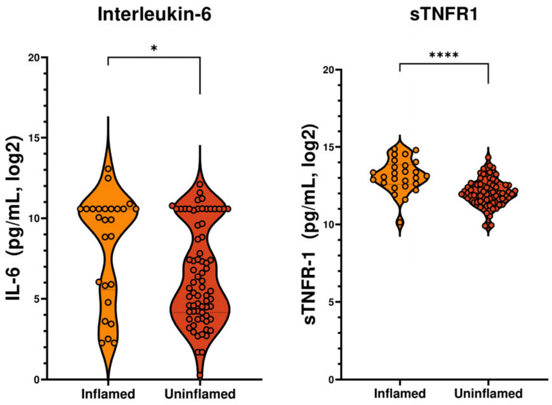
Interleukin-6
*
IL-6 (pg/mL, log2)
20
15
10
5
0
Inflamed
Uninflamed
sTNFR1
****
sTNFR-1 (pg/mL, log2)
20
15
10
5
0
Inflamed
Uninflamed

Figure E9:

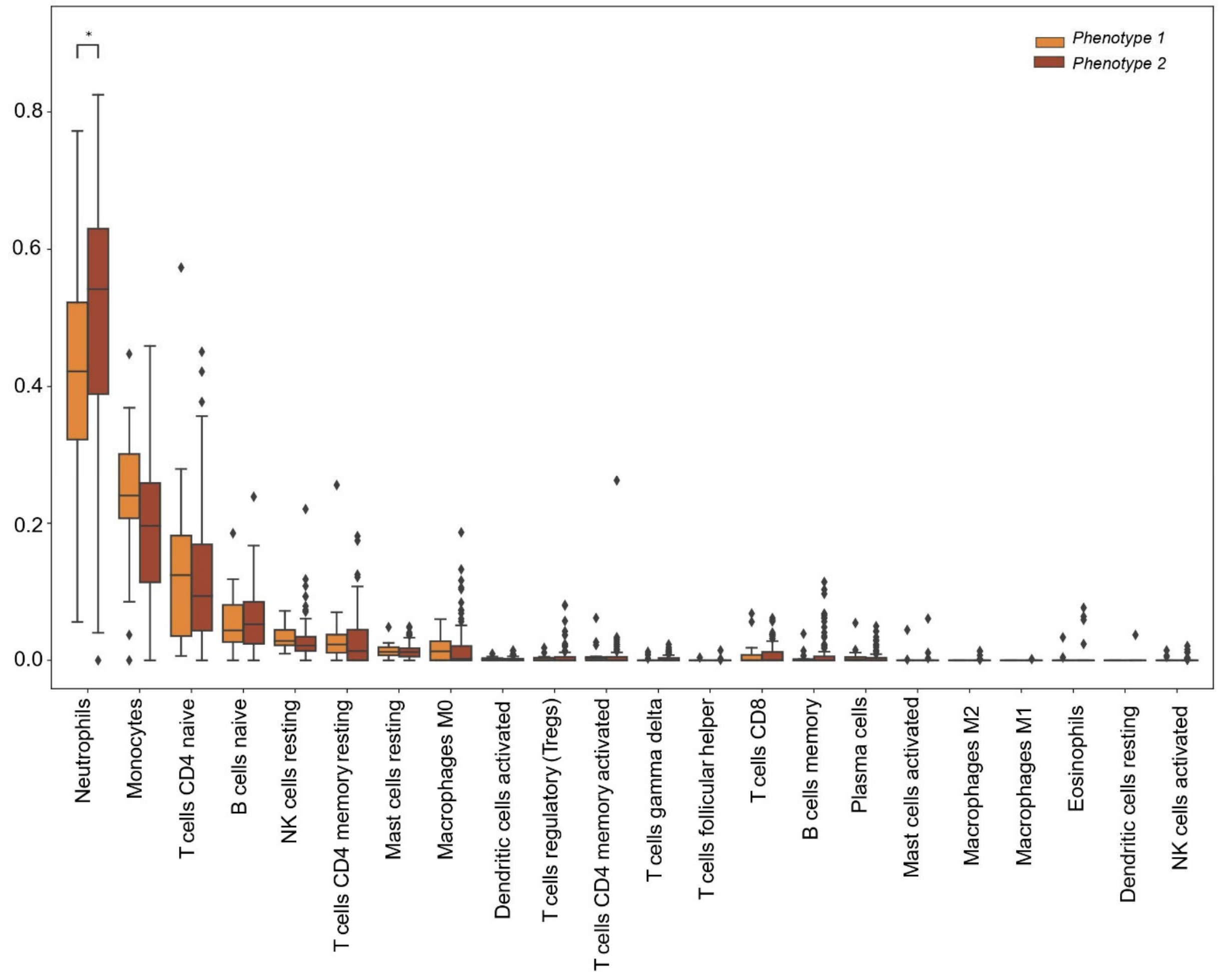
*
Phenotype 1
Phenotype 2
0.8
0.6
0.4
0.2
0.0
Neutrophils
Monocytes
T cells CD4 naive
B cells naive
NK cells resting
T cells CD4 memory resting
Mast cells resting
Macrophages M0
Dendritic cells activated
T cells regulatory (Tregs)
T cells CD4 memory activated
T cells gamma delta
T cells follicular helper
T cells CD8
B cells memory
Plasma cells
Mast cells activated
Macrophages M2
Macrophages M1
Eosinophils
Dendritic cells resting
NK cells activated

Figure E10:

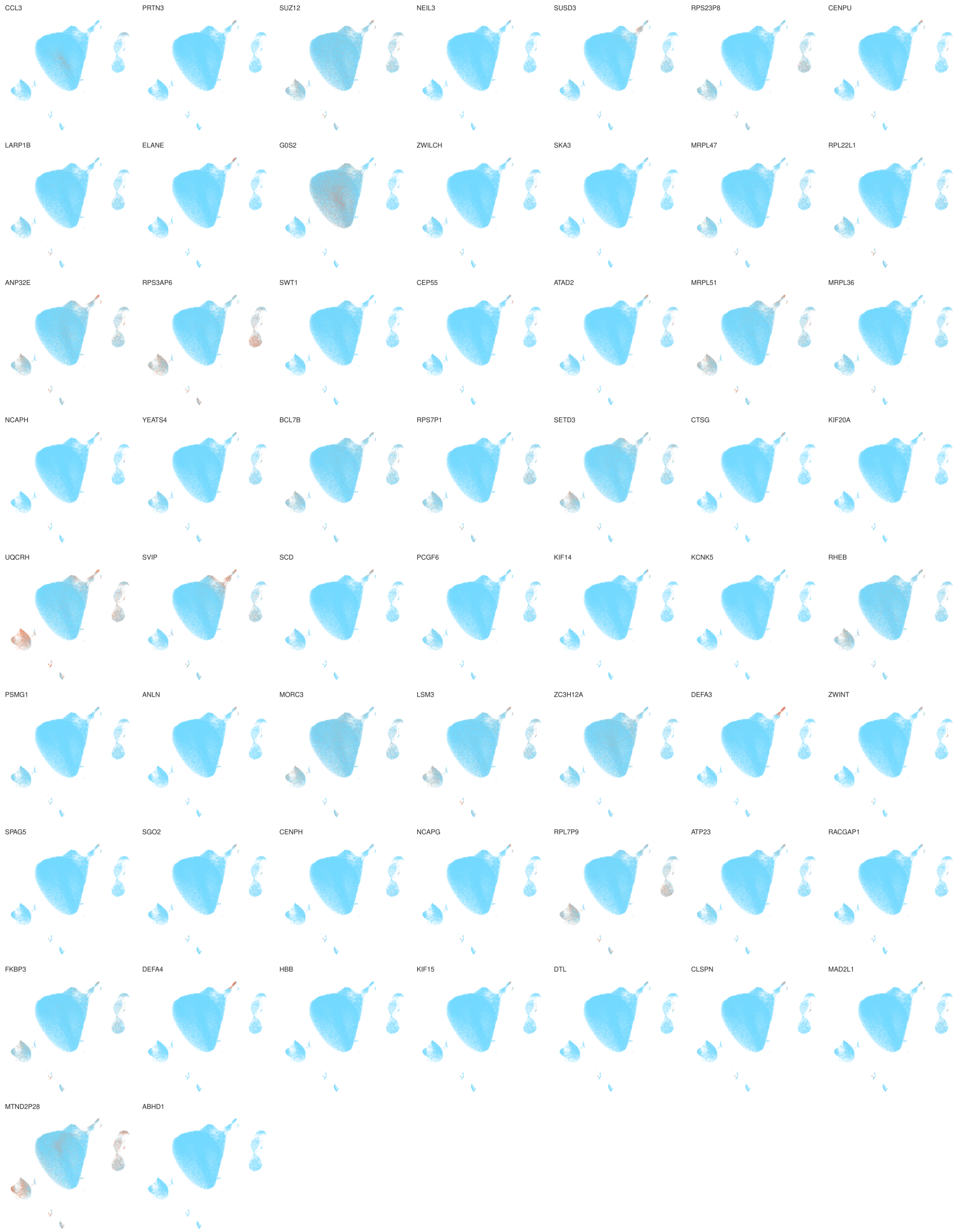
CCL3
PRTN3
SUZ12
NEIL3
SUSD3
RPS23P8
CENPU
LARP1B
ELANE
G0S2
ZWILCH
SKA3
MRPL47
RPL22L1
ANP32E
RPS3AP6
SWT1
CEP55
ATAD2
MRPL51
MRPL36
NCAPH
YEATS4
BCL7B
RPS7P1
SETD3
CTSG
KIF20A
UQCRH
SVIP
SCD
PCGF6
KIF14
KCNK5
RHEB
PSMG1
ANLN
MORC3
LSM3
ZC3H12A
DEFA3
ZWINT
SPAG5
SGO2
CENPH
NCAPG
RPL7P9
ATP23
RACGAP1
FKBP3
DEFA4
HBB
KIF15
DTL
CLSPN
MAD2L1
MTND2P28
ABHD1

Figure E11:

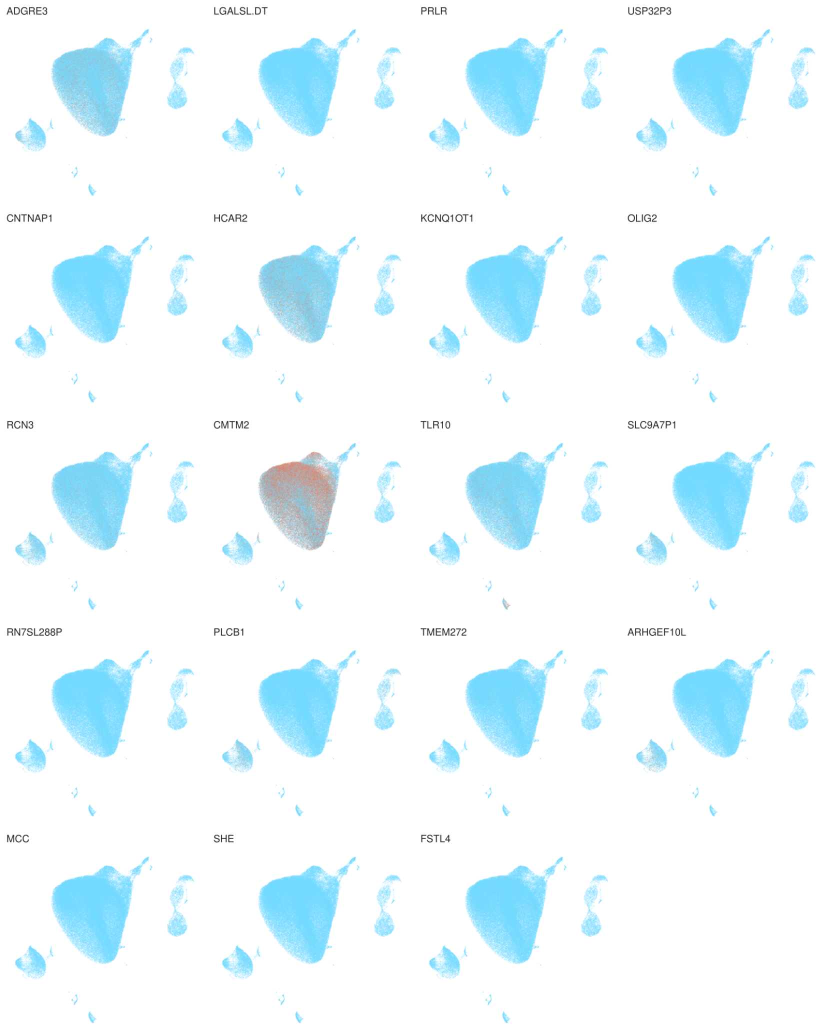
ADGRE3
LGALSL.DT
PRLR
USP32P3
CNTNAP1
HCAR2
KCNQ1OT1
OLIG2
RCN3
CMTM2
TLR10
SLC9A7P1
RN7SL288P
PLCB1
TMEM272
ARHGEF10L
MCC
SHE
FSTL4

References:

1. Fleming S, Thompson M, Stevens R, Heneghan C, Plüddemann A, Maconochie I, et al. Normal ranges of heart rate and respiratory rate in children from birth to 18 years of age: a systematic review of observational studies. The Lancet. 2011 Mar 19;377(9770):1011–8.

2. National High Blood Pressure Education Program Working Group on High Blood Pressure in Children and Adolescents. The fourth report on the diagnosis, evaluation, and treatment of high blood pressure in children and adolescents. Pediatrics. 2004 Aug;114(2 Suppl 4th Report):555–76.

3. McIntosh AM, Tong S, Deakyne SJ, Davidson JA, Scott HF. Validation of the Vasoactive-Inotropic Score in Pediatric Sepsis*. Pediatr Crit Care Med. 2017 Aug;18(8):750–7.

4. Wong HR, Cvijanovich NZ, Anas N, Allen GL, Thomas NJ, Bigham MT, et al. PERSEVERE-II: Redefining the pediatric sepsis biomarker risk model with septic shock phenotype. Crit Care Med. 2016 Nov;44(11):2010–7.

5. Wong HR, Caldwell JT, Cvijanovich NZ, Weiss SL, Fitzgerald JC, Bigham MT, et al. Prospective clinical testing and experimental validation of the Pediatric Sepsis Biomarker Risk Model. Sci Transl Med [Internet]. 2019 Nov 13 [cited 2021 Mar 15];11(518). Available from: https://www.ncbi.nlm.nih.gov/pmc/articles/PMC7720682/

6. Atreya MR, Cvijanovich NZ, Fitzgerald JC, Weiss SL, Bigham MT, Jain PN, et al. Integrated PERSEVERE and endothelial biomarker risk model predicts death and persistent MODS in pediatric septic shock: a secondary analysis of a prospective observational study. Critical Care. 2022 Jul 11;26(1):210.

7. Atreya MR, Cvijanovich NZ, Fitzgerald JC, Weiss SL, Bigham MT, Jain PN, et al. Prognostic and predictive value of endothelial dysfunction biomarkers in sepsis-associated acute kidney injury: risk-stratified analysis from a prospective observational cohort of pediatric septic shock. Crit Care. 2023 Jul 3;27(1):260.

8. Bießmann F, Rukat T, Schmidt P, Naidu P, Schelter S, Taptunov A, et al. DataWig: Missing Value Imputation for Tables.

9. Kwok AJ, Allcock A, Ferreira RC, Cano-Gamez E, Smee M, Burnham KL, et al. Neutrophils and emergency granulopoiesis drive immune suppression and an extreme response endotype during sepsis. Nat Immunol. 2023 May;24(5):767–79.

10. Zheng H, Rao AM, Dermadi D, Toh J, Murphy Jones L, Donato M, et al. Multi-cohort analysis of host immune response identifies conserved protective and detrimental modules associated with severity across viruses. Immunity. 2021 Apr 13;54(4):753-768.e5.

11. Wong HR, Cvijanovich NZ, Anas N, Allen GL, Thomas NJ, Bigham MT, et al. Developing a clinically feasible personalized medicine approach to pediatric septic shock. Am J Respir Crit Care Med. 2015 Feb 1;191(3):309–15.
